# Mosaic Ends Tagmentation (METa) Assembly for Highly Efficient Construction of Functional Metagenomic Libraries

**DOI:** 10.1128/mSystems.00524-21

**Published:** 2021-06-29

**Authors:** Terence S. Crofts, Alexander G. McFarland, Erica M. Hartmann

**Affiliations:** a Department of Molecular Biosciences, Northwestern University, Evanston, Illinois, USA; b Department of Civil and Environmental Engineering, Northwestern University, Evanston, Illinois, USA; Wageningen University

**Keywords:** antibiotic resistance, beta-lactamases, colistin, functional metagenomics, microbiome, nourseothricin, rRNA methyltransferase, shotgun cloning, streptothricin acetyltransferase, tetracyclines

## Abstract

Functional metagenomic libraries, physical bacterial libraries which allow the high-throughput capture and expression of microbiome genes, have been instrumental in the sequence-naive and cultivation-independent exploration of metagenomes. However, preparation of these libraries is often limited by their high DNA input requirement and their low cloning efficiency. Here, we describe a new method, mosaic ends tagmentation (METa) assembly, for highly efficient functional metagenomic library preparation. We applied tagmentation to metagenomic DNA from soil and gut microbiomes to prepare DNA inserts for high-throughput cloning into functional metagenomic libraries. The presence of mosaic end sequences in the resulting DNA fragments synergized with homology-based assembly cloning to result in a 300-fold increase in cloning efficiency compared to traditional blunt-cloning-based protocols. We show that compared to published libraries prepared by state-of-the-art protocols, METa assembly is on average ca. 20- to 200-fold more efficient and can prepare gigabase-sized libraries with as little as 200 ng of input DNA. We show the usefulness of METa assembly first by using a normative 5-μg mass of soil metagenomic DNA to prepare a 700-Gb library that allowed us to discover novel nourseothricin resistance genes and a potentially new mode of resistance to this antibiotic and second by using only 300 ng of goose fecal metagenomic DNA to prepare a 27-Gb library that captured numerous tetracycline and colistin resistance genes. METa assembly provides a streamlined, flexible, and efficient method for preparing functional metagenomic libraries, enabling new avenues of genetic and biochemical research into low-biomass or scarce microbiomes.

**IMPORTANCE** Medically and industrially important genes can be recovered from microbial communities by high-throughput sequencing, but precise annotation is often limited to characterized genes and their relatives. Cloning a metagenome *en masse* into an expression host to produce a functional metagenomic library, directly connecting genes to functions, is a sequence-naive and cultivation-independent method to discover novel genes. The process of preparing these libraries is DNA greedy and inefficient, however. Here, we describe a library preparation method that is an order of magnitude more efficient and less DNA greedy. This method is consistently efficient across libraries prepared from cultures, a soil microbiome, and a goose fecal microbiome and allowed us to discover new antibiotic resistance genes and mechanisms. This library preparation method will potentially allow the functional metagenomic exploration of microbiomes that were previously off limits due to their rarity or low microbial biomass, such as biomedical swabs or exotic samples.

## INTRODUCTION

The widespread adoption of high-throughput DNA sequencing technology has resulted in a new and deserved appreciation for the genetic diversity present in microbial communities, also called microbiomes (1). Projects studying the functional potential of microbiomes have shown that this genetic diversity translates into enormous biochemical diversity (2–6). However, accurately linking novel genes from microbial community genetic material (the metagenome) to biochemical activity remains difficult due to limitations in gene prediction and functional annotation. Direct observation of biochemical function *in vitro* or phenotype *in vivo* remains the gold standard of functional assignment as a result (7, 8).

**FIG 1:**
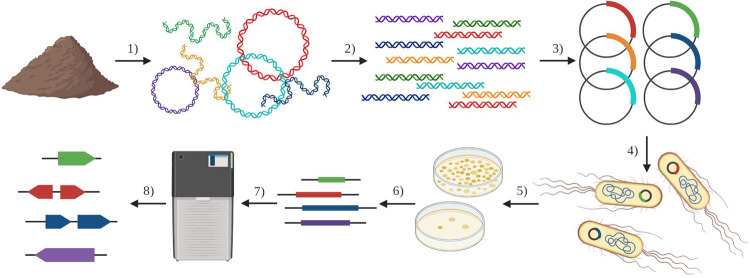
Functional metagenomic library pipeline. The general pipeline for the creation and use of functional metagenomic libraries to capture and discover genes from metagenomes. (Step 1) Extraction of metagenomic DNA from a microbiome (e.g., soil or fecal samples). (Step 2) Fragmentation of metagenomic DNA to desired size range (e.g., by sonication, restriction enzyme digestion, or tagmentation). (Step 3) Cloning of fragments into expression vectors following size selection (e.g., by blunt ligation or homology-based assembly. (Step 4) Transformation *en masse* of vectors into an expression host (e.g., E. coli) to create functional metagenomic library. (Step 5) Functional selection or screen of library (e.g., on antibiotics to select for resistance). (Step 6) Amplification of selected inserts using vector-specific primers. (Step 7) High-throughput sequencing of selected metagenomic amplicons (e.g., by Illumina or PacBio technologies). (Step 8) Annotation of sequenced amplicons to link novel genes with selected/screened function (e.g., discovery of novel antibiotic resistance genes). (Created in BioRender.com.)

One method that unites the culture- and sequence-independence of high-throughput sequencing with the functional observations that result from cloning and expression studies is functional metagenomics. Functional metagenomics relies upon the construction of metagenomic libraries in which a portion of a microbiome’s metagenome is captured in a bacterial artificial chromosome, fosmid, or plasmid library and housed in an expression host, often Escherichia coli (2) (Fig. 1). This technique allows function to be linked directly to genes without requiring laboratory growth of the originating organisms or prior knowledge of the target gene sequence. Functional metagenomic libraries have been used to bioprospect for novel bioactive compounds (9; for reviews, see references 3 and 10), novel enzymes of potential interest to industry (11, 12), and enzymes useful in the production of biofuels (13, 14; for reviews, see references 15–17) and have been created using metagenomic DNA from environments as varied as soils, adult and infant fecal samples, sewage and wastewater effluent, and animal samples (18–35). One particularly successful application of functional metagenomic libraries has been in the identification of antimicrobial resistance genes (7, 36) that would have eluded identification by sequencing due to their low predicted amino acid identity.

The basic steps for creating a functional metagenomic library consist of metagenomic DNA extraction, DNA fragmentation, cloning of fragments into a vector, and transfer of the plasmid library into an expression host (6, 36) (Fig. 1). Small insert functional metagenomic selections and screens are a popular form of this method and are used to discover individual genes or small operons. The libraries used in these experiments contain inserts between 1 kb and 10 kb in length and often use sonication or acoustic shearing to fragment metagenomic DNA, followed by blunt cloning of inserts into an expression plasmid (18, 21, 25, 26, 33, 37) (large insert libraries use fosmid cloning methods instead). These two steps, physical fragmentation and blunt cloning (steps 2 and 3 in Fig. 1), greatly lower the potential efficiency of functional metagenomic library creation, often necessitating high input DNA mass (e.g., 10 μg [37]).

Like functional metagenomic libraries, shotgun sequencing libraries have, until recently, relied largely upon physical methods for DNA fragmentation and have similarly required substantial input DNA mass. In contrast, transposase enzymatic DNA fragmentation (38, 39) (tagmentation, known commercially as Nextera) produces DNA fragments using transposomes (complexes of transposase enzyme with an oligonucleotide cargo) that create mostly random (38, 40) double-stranded DNA breaks by insertion of their oligonucleotide cargo (38). This method substantially decreases costs and input DNA mass requirements (38–43) but has not yet been applied to the preparation of functional metagenomic libraries. Similarly, other enzymatic methods have been applied to prepare inserts for functional metagenomic libraries, including the use of restriction enzyme endonucleases to digest metagenomic DNA (44). However, this method is associated with shortcomings, including sensitivity to DNA methylation state and nonrandom cutting. More recently, another enzyme used in the preparation of high-throughput sequencing libraries, fragmentase from New England Biolabs (NEB), has been successfully used to prepare small insert functional metagenomic libraries (45).

**FIG 2:**
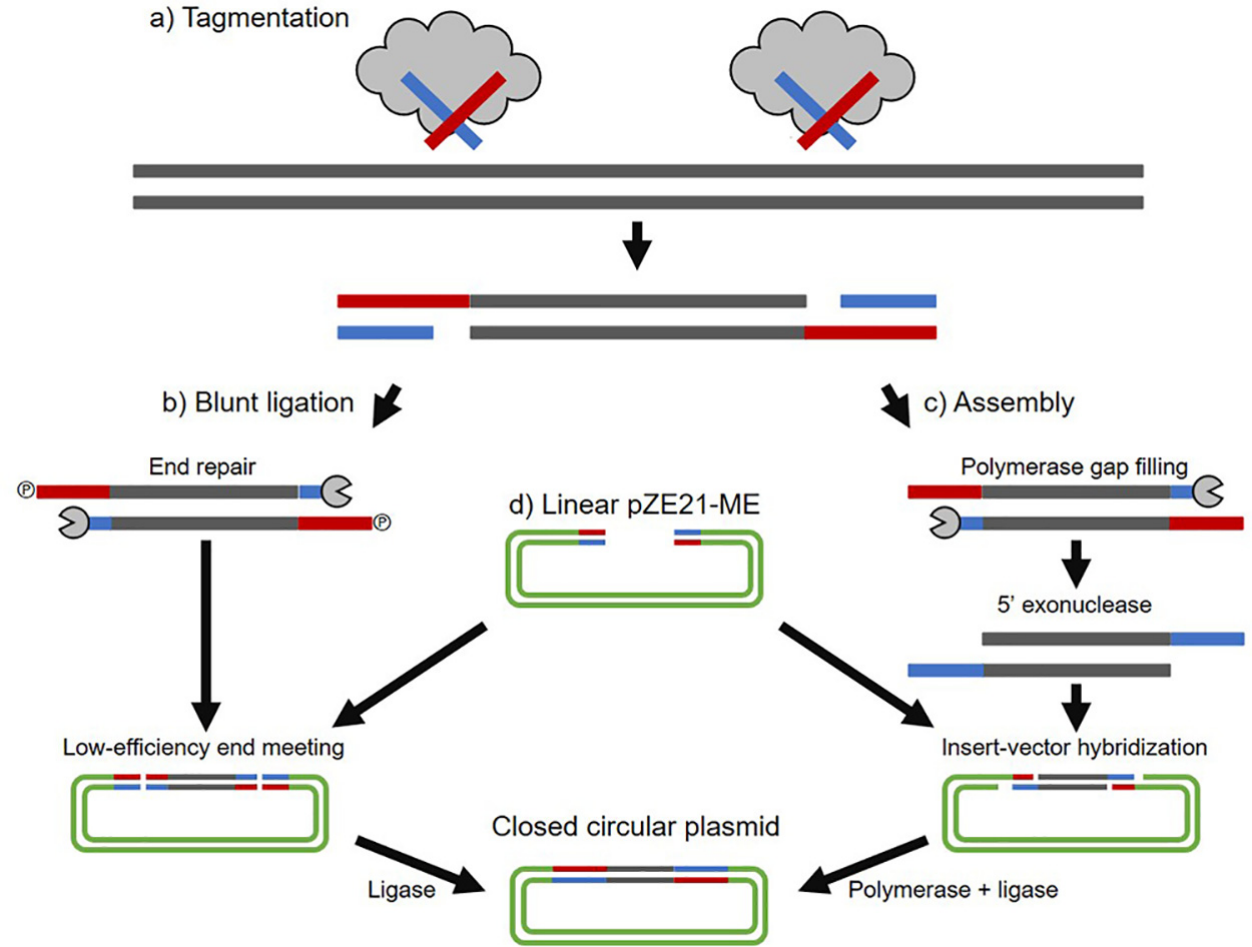
Blunt cloning protocol compared to METa assembly. (a) Transposome (transposase enzyme loaded with mosaic end oligonucleotides) fragments DNA with 5′ mosaic end oligonucleotides. Tagmentation inserts can be used as input for both methods. (b) Blunt ligation. 5′ overhangs must be resolved by gap filling and phosphorylation using end-repair enzyme mixes. Blunt-ended inserts can be ligated into blunt-ended vector. (c) METa assembly. 5′ overhangs must be resolved by DNA polymerase gap filling. The assembly enzyme mix includes 5′ exonuclease to create 3′ overhangs which hybridize with target pZE21-ME. DNA polymerase fills in gaps, and ligase seals nicks. (d) pZE21-ME is prepared and linearized by inverse PCR and is compatible with either pipeline.

We hypothesized that tagmentation reactions could be used in the preparation of functional metagenomic libraries (Fig. 2a), likely dramatically decreasing input DNA requirements compared to acoustic shearing methods while avoiding shortcomings associated with restriction endonucleases. Tagmentation, unlike fragmentase treatment, also results in the incorporation of transposome oligonucleotides on the ends of each piece of fragmented DNA. We hypothesized that incorporation of these sequences on the ends of inserts could allow us to use homology-based DNA assembly protocols (e.g., Gibson assembly [46], etc.) (Fig. 2c) in place of blunt ligation (Fig. 2b). We hypothesized that incorporation of matching sequences in an expression vector (Fig. 2d) would allow the vector to capture inserts using extensive base-pairing, leading to significantly increased efficiency in library preparation.

Here, we report our testing of these hypotheses and the development, validation, and application of a new general method for functional metagenomic library preparation that we are calling mosaic ends tagmentation (METa) assembly. Our method takes advantage of the so-far-unexplored synergy between tagmentation and assembly cloning to produce functional metagenomic libraries with up to 270-fold more efficiency and 25-fold-reduced input DNA mass requirements compared to current methods for small-insert functional metagenomic library preparation. METa assembly has the potential to greatly improve and expand the field of bioprospecting, catalyzing the discovery of novel microbial chemistry from genetically diverse microbiomes.

## RESULTS

### Strategies to improve functional metagenomic library preparation efficiency.

Our first strategy to increase functional metagenomic library preparation efficiency was to replace acoustic fragmentation with transposase-based tagmentation (Fig. 2a). In theory, this would allow lower input DNA mass and obviate expensive capital equipment needed for the sonication-based blunt-ligation cloning protocol while retaining near-random fragmentation (38). Our second strategy to increase efficiency was to replace blunt-ligation cloning with homology-based seamless assembly cloning, taking advantage of the fact that tagmentation-produced fragments have a known DNA sequence on their ends (Fig. 2a). One assembly option that we hypothesized would be compatible was NEBuilder HiFi DNA assembly from New England Biolabs, which functions similarly to Gibson assembly (46). This method requires overlap regions with melting temperatures greater than 50°C, which is compatible with the 19-bp mosaic end sequence favored by Tn*5* transposases (an estimated melting temperature of 52°C by the 2AT + 4GC rule) (47). NEBuilder HiFi and Gibson assembly use 5′ exonucleases to produce 3′ overhangs (Fig. 2c). Because tagmentation results in covalent addition of mosaic end oligonucleotides to only the 5′ ends of DNA fragments, 5′ exonuclease activity would effectively erase the mosaic end sequence from the inserts. Nextera tagmentation protocols overcome this obstacle by including a brief DNA polymerase gap-filling reaction that would be applicable to our protocol as well. The resulting DNA would be mixed with the NEBuilder HiFi assembly master mix, resulting in 3′ overhangs able to hybridize to complementary sequences on linearized vector. Following hybridization, NEBuilder HiFi assembly master mix polymerase and ligase fill gaps and ligate nicks, respectively, to produce a covalently sealed construct for transformation (Fig. 2d).

### Tagmentation conditions to target 1-kb to 10-kb inserts.

**FIG 3:**
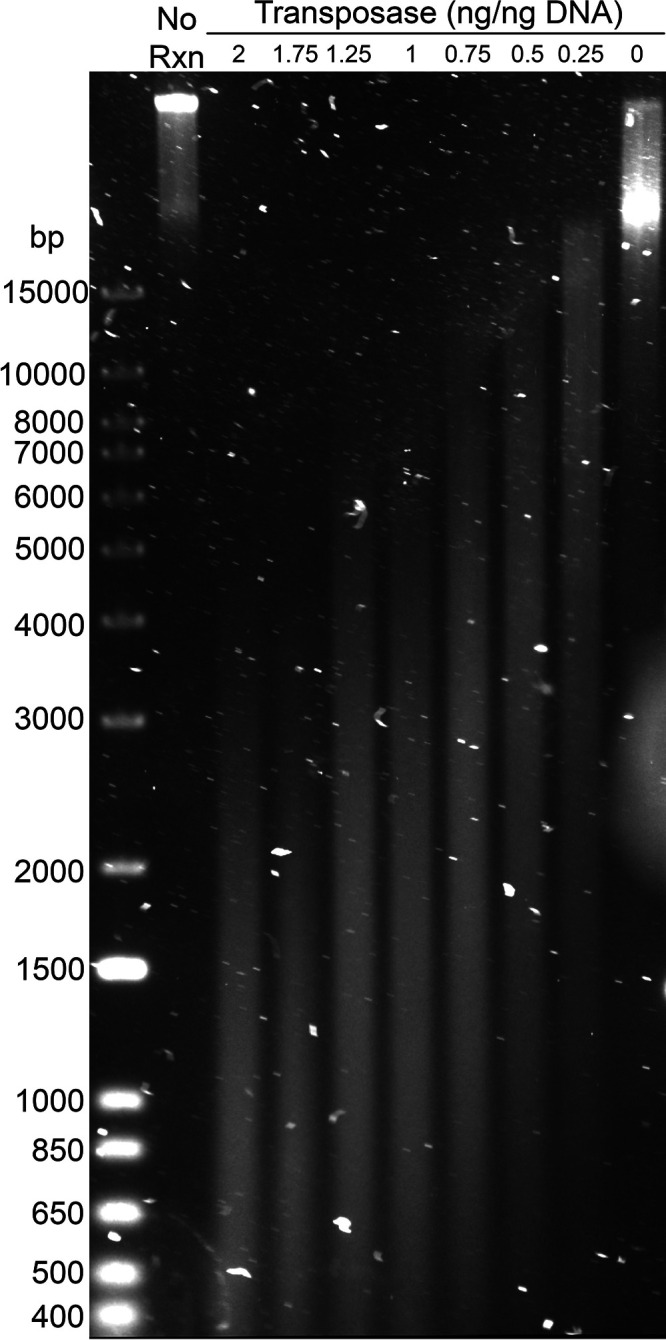
Transposome to DNA ratios can be adjusted to target 1-kb to 10-kb fragments. High-molecular-weight genomic DNA (Rxn, reaction) was incubated with transposome at concentrations from 0 ng/ng of DNA up to 2 ng/ng DNA. The resulting fragments were analyzed by pulsed-field agarose gel electrophoresis.

To begin testing these strategies we first investigated if tagmentation could be used to prepare metagenomic or genomic DNA in fragments roughly 1 kb to 5 kb in length appropriate for small insert functional metagenomic libraries (i.e., capable of containing bacterial open reading frames [ORFs]). We used in-house-purified transposase enzyme (Text S1 and S2) and high-molecular-weight genomic DNA (measured at ∼70 kb) isolated from two penicillin-catabolizing bacteria, ABC07 (Pseudomonas sp. strain PE-S1G-1) and ABC10 (*Pandoraea* sp. strain PE-S2T-3) (48–50) as input for these test reactions. While tagmentation is usually used to create ∼200-bp DNA fragments for sequencing on the Illumina platform, we were able to alter this by adjusting the ratio of transposome to input. Using 0.5 ng of transposome per ng of target DNA yielded a fragmentation pattern centered around 2.5 kb (Fig. 3).

10.1128/mSystems.00524-21.1 TEXT S1 Description of the expression and purification of Tn5 transposase and initial activity tests. Download Text S1, DOCX file, 0.02 MB. Copyright © 2021 Crofts et al. 2021 Crofts et al. https://creativecommons.org/licenses/by/4.0/ This content is distributed under the terms of the Creative Commons Attribution 4.0 International license.

10.1128/mSystems.00524-21.2 TEXT S2 Results of transposase expression, purification, and testing. Download Text S2, DOCX file, 0.02 MB. Copyright © 2021 Crofts et al. 2021 Crofts et al. https://creativecommons.org/licenses/by/4.0/ This content is distributed under the terms of the Creative Commons Attribution 4.0 International license.

### Comparison of blunt ligation and assembly for library preparation using tagmented DNA input.

We hypothesized that inserts prepared by tagmentation could be compatible with both blunt ligation and assembly-based methods of functional metagenomic library preparation (Fig. 2b and c). To test this hypothesis, we combined high-molecular-weight DNA from two bacterial strains of interest to us: Pseudomonas sp. strain PE-S1G-1 (ABC07) and *Pandoraea* sp. strain PE-S2T-3 (ABC10). The pooled DNA was fragmented by tagmentation (Fig. S1a) and size selected for fragments between approximately 1 kb and 8 kb. The resulting stock of mosaic end 5′-tagged DNA fragments was used as input for triplicate blunt ligation and triplicate assembly cloning reactions to prepare functional metagenomic libraries. After cloning inserts into vector (by assembly or blunt ligation), we electroporated the entirety of each purified reaction mixture into E. coli cells, plated dilutions of the recovered cells to determine titers, and inoculated overnight cultures to amplify each library. The following day, we used colony PCR to find the average insert size and proportion of colonies with an insert (as opposed to empty vectors) (Fig. S1b).

10.1128/mSystems.00524-21.5 FIG S1 Representative DNA tagmentation and colony PCR agarose gels. (a) Mixed ABC07, ABC10 genomic DNA was tagmented and purified from agarose gel. DNA fragments were excised from ∼1264 bp to ∼8454 bp. (b) Colony PCR was performed on triplicate assembly and blunt cloning libraries to determine average insert size and proportion of plasmids containing inserts. Lanes 1, 25, 45, and 69, DNA ladders with 5.0-kb, 1.5-kb, and 0.5-kb bands indicated. Lanes 2 to 24 and 26 to 44, colonies from three replicate assemblies using NEBuilder HiFi (13 colonies per replicate; lanes 15, 30, and 44 are from negative-control sham colonies). Lanes 46 to 68 and 70 to 88, colonies from triplicate blunt-ligation cloning reactions (13 colonies per replicate; lanes 59, 74, and 88 are from negative-control sham colonies). Reactions resulting in 500-bp amplicons indicate carriage of a vector without an insert. Reactions resulting in no visible band indicate technical failure of the colony PCR. Download FIG S1, JPG file, 3.7 MB. Copyright © 2021 Crofts et al. 2021 Crofts et al. https://creativecommons.org/licenses/by/4.0/ This content is distributed under the terms of the Creative Commons Attribution 4.0 International license.

**FIG 4:**
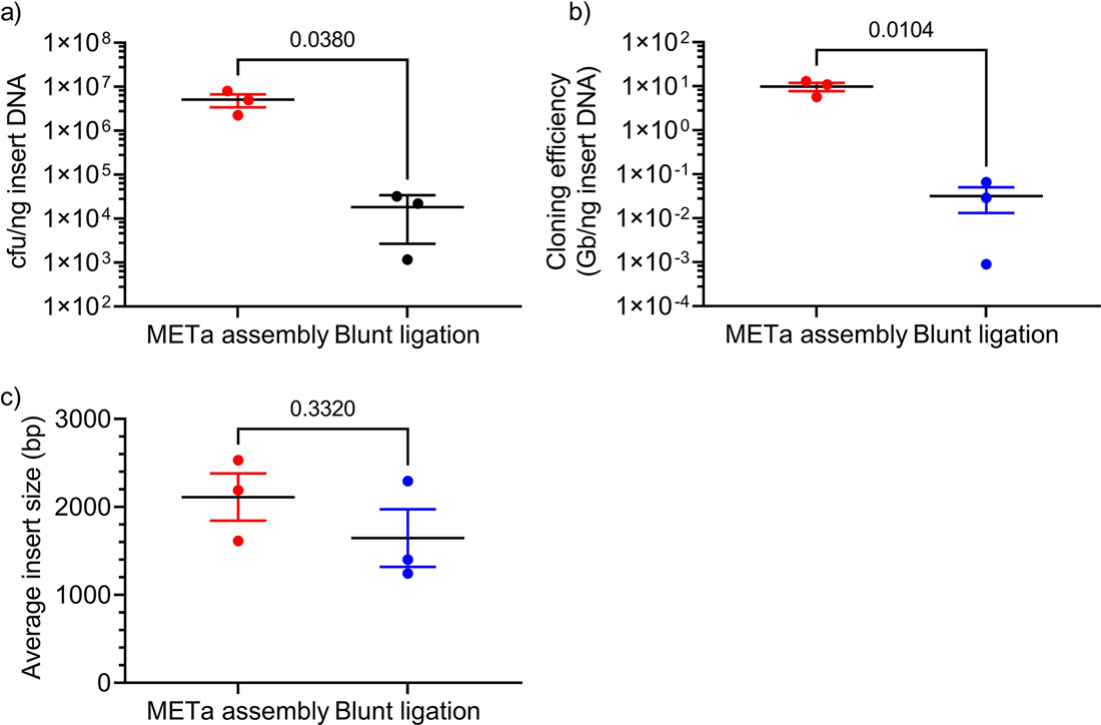
Libraries created by METa assembly are larger than those created by blunt ligation. Both sets of triplicate metagenomic library assembly/cloning reactions used the same input DNA and were compared using unpaired two-tailed t tests (*P* values shown). Error bars represent standard errors from 3 experiments. (a) Culture titers of recovered cells posttransformation normalized to insert DNA mass used in assembly or cloning. (b) Cloning efficiency (gigabases per nanogram of insert DNA used for assembly or blunt ligation). (c) Average insert size determined by colony PCR, excluding colonies containing empty vector constructs.

We found that METa assembly resulted in significantly higher titers of transformed cells per nanogram of insert DNA used during the cloning step of library preparation (Fig. 4a) (∼276-fold greater than the blunt-ligation libraries; *P = *0.038) and significantly greater cloning efficiency (library size normalized to DNA inserts used in the cloning step) (Fig. 4b) (∼310-fold greater than the blunt-ligation libraries; *P = *0.0104). Average insert size (Fig. 4c) did not appear to differ significantly between methods (*P* = 0.3320) (summarized in Table S1B). Colonies containing empty vectors occurred more frequently following blunt-ligation reactions (4/13, 6/12, and 5/10 by colony PCR) than colonies from METa assembly reactions (0/11, 0/12, and 0/13 by colony PCR) (Fig. S1b), providing a useful metric by which to measure the robustness of assembly versus blunt-ligation libraries.

10.1128/mSystems.00524-21.3 TABLE S1 Oligonucleotides and library summaries. (A) Oligonucleotides used in the study. (B) Details of triplicate ABC07/ABC10 blunt cloning and assembly libraries. (C) Details of literature and METa assembly libraries. Download Table S1, XLSX file, 0.01 MB. Copyright © 2021 Crofts et al. 2021 Crofts et al. https://creativecommons.org/licenses/by/4.0/ This content is distributed under the terms of the Creative Commons Attribution 4.0 International license.

**FIG 5:**
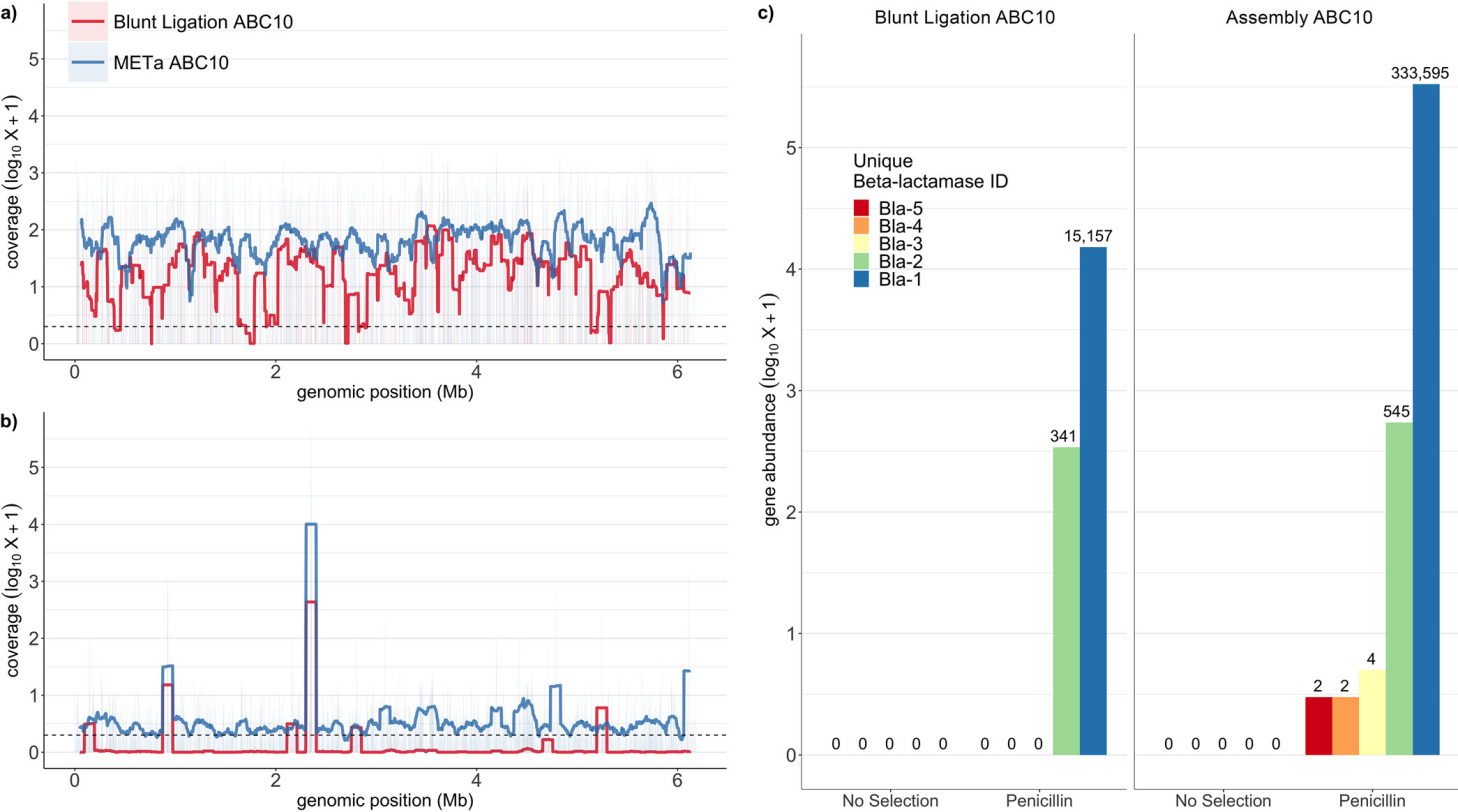
Assembly and blunt-ligation library coverage of ABC010 genome with and without penicillin selection. (a) Nucleotide depth of coverage for ABC10 genome by functional metagenomic library prepared by assembly (blue) or blunt ligation (red). Coverage is smoothed to a 1-kb resolution. (b) As for panel a, but sequenced libraries were first subjected to selection on agar plates containing 1 mg/ml penicillin. (c) Gene abundance in post-penicillin selection reads for each of five predicted ABC10 β-lactamase genes that had read numbers of >0. NCBI accession numbers for Bla-1 to Bla-5, respectively, are WP_087722475.1, WP_087721859.1, WP_140413467.1, WP_087721948.1, and WP_087721885.1.

After establishing that assembly cloning results in much larger libraries than blunt-ligation cloning, we next asked if genomic coverage was similar across methods. We sequenced between 355 and 831 random colonies from each of the six ABC07/ABC10 multigenomic libraries. Each library was plated to give approximately 1,000 colonies based on prior titers, resulting in an average of approximately 600 colonies collected (ca. 1,562 total colonies from blunt-ligation plates; 2,008 total colonies from assembly plates). We extracted plasmids from the collected colonies and pooled each set of triplicate libraries into a single pool. These two pools were used as templates in a limited PCR to amplify inserts (Fig. S2), which were submitted for long-read sequencing on the PacBio Sequel II platform. The resulting reads were mapped back onto published ABC07 and ABC10 genomes (48), and the nucleotide coverage of each library for each genome was calculated and smoothed to a 1-kb resolution. We found qualitatively good agreement between assembly and blunt-ligation library coverage of both the ABC10 (Fig. 5a) and ABC07 (Fig. S3a) genomes.

10.1128/mSystems.00524-21.6 FIG S2 Amplicons from functional metagenomic library minipreps for sequencing. From left to right: L (ladder); (1) pooled triplicate ABC07/10 library prepared by METa assembly, no antibiotics; (2) same as 1, following selection on 1 mg/ml penicillin; (3) pooled triplicate ABC07/10 library prepared by blunt ligation, no antibiotics; (4) same as 3, following selection on 1 mg/ml penicillin; (5) 5-μg soil microbiome library following selection on 64 μg/ml nourseothricin; (6) 300 ng goose fecal microbiome library following selection on 8 μg/ml tetracycline; (7) same as 6-TSC but with selection on 4 μg/ml colistin. Download FIG S2, JPG file, 0.2 MB. Copyright © 2021 Crofts et al. 2021 Crofts et al. https://creativecommons.org/licenses/by/4.0/ This content is distributed under the terms of the Creative Commons Attribution 4.0 International license.

10.1128/mSystems.00524-21.7 FIG S3 Assembly and blunt-ligation library coverage of ABC07 genome with and without penicillin selection. (a) Nucleotide depth of coverage for ABC07 genome by functional metagenomic library prepared by assembly (blue) or blunt ligation (red). Coverage is smoothed to a 1-kb resolution. (b) As for panel a, but sequenced libraries were first subjected to selection on agar plates containing 1 mg/ml penicillin. (c) Gene abundance in post-penicillin selection reads for each of six predicted ABC07 β-lactamase genes that had read numbers of >0. NCBI accession numbers for Bla-1 to Bla-6, respectively, are WP_087694996.1, WP_003211977.1, WP_003216184.1, WP_087694154.1, WP_008436310.1, WP_003207471.1. Download FIG S3, JPG file, 1.7 MB. Copyright © 2021 Crofts et al. 2021 Crofts et al. https://creativecommons.org/licenses/by/4.0/ This content is distributed under the terms of the Creative Commons Attribution 4.0 International license.

In order to verify the functional aspect of our functional metagenomic libraries, we next performed triplicate selections for growth in the presence of 1 mg/ml penicillin. This concentration, about 10-fold higher than the E. coli DH10B minimal inhibitory concentration (MIC), was chosen based on the high penicillin resistance of strains ABC07 and ABC10. Triplicate libraries were plated with the goal of reaching 10-fold coverage of each library, resulting in denser plating for the much larger METa assembly triplicate libraries. Colonies from each triplicate plating were collected and pooled for plasmid purification and PCR amplification of inserts for sequencing (Fig. S2). Sequencing for both library pools was dominated by reads mapping to one genomic region (ABC07) (Fig. S3b) or two genomic regions (ABC10) (Fig. 5b) of the donor organism. In each case, these regions corresponded to predicted β-lactamase genes (Fig. 5c; Fig. S3c).

### Preparation of a large soil metagenomic library by METa assembly and discovery of novel resistance genes.

We next prepared a functional metagenomic library by METa assembly for direct comparison to published libraries prepared by blunt ligation. A common DNA input quantity for preparation of a single functional metagenomic library in the acoustic fragmentation/blunt-ligation workflow is ∼5 μg (21, 28, 35, 37, 51, 52). In order to compare METa assembly to the broader literature, we performed a tagmentation reaction on 5 μg of soil metagenomic DNA. Following size selection and polymerase gap filling, we retained 1.122 μg of mosaic end-containing inserts ready for assembly. Because this quantity of DNA falls well outside the recommended capacity of NEBuilder HiFi reactions, we first assembled 16.6% (175 ng) of the total in a trial reaction. Transformation of the purified assembly reaction resulted in a 162.0 ± 22.4-Gb library (7.8 × 10^7^ unique clones; average insert size, 2.077 kb; no empty vectors). After scaling up assembly and transformation, the remaining inserts were used to prepare a 529.2 ± 60.2-Gb library (2.85 × 10^8^ unique clones; average insert size, 1.856 kb; no empty vectors). When library counts and colony PCR data were combined, these libraries together were calculated to form a 703.2 ± 72.4-Gb library from 5 μg of input DNA (no empty vectors in 46 colony PCR amplicons) (Table S1C).

**FIG 6:**
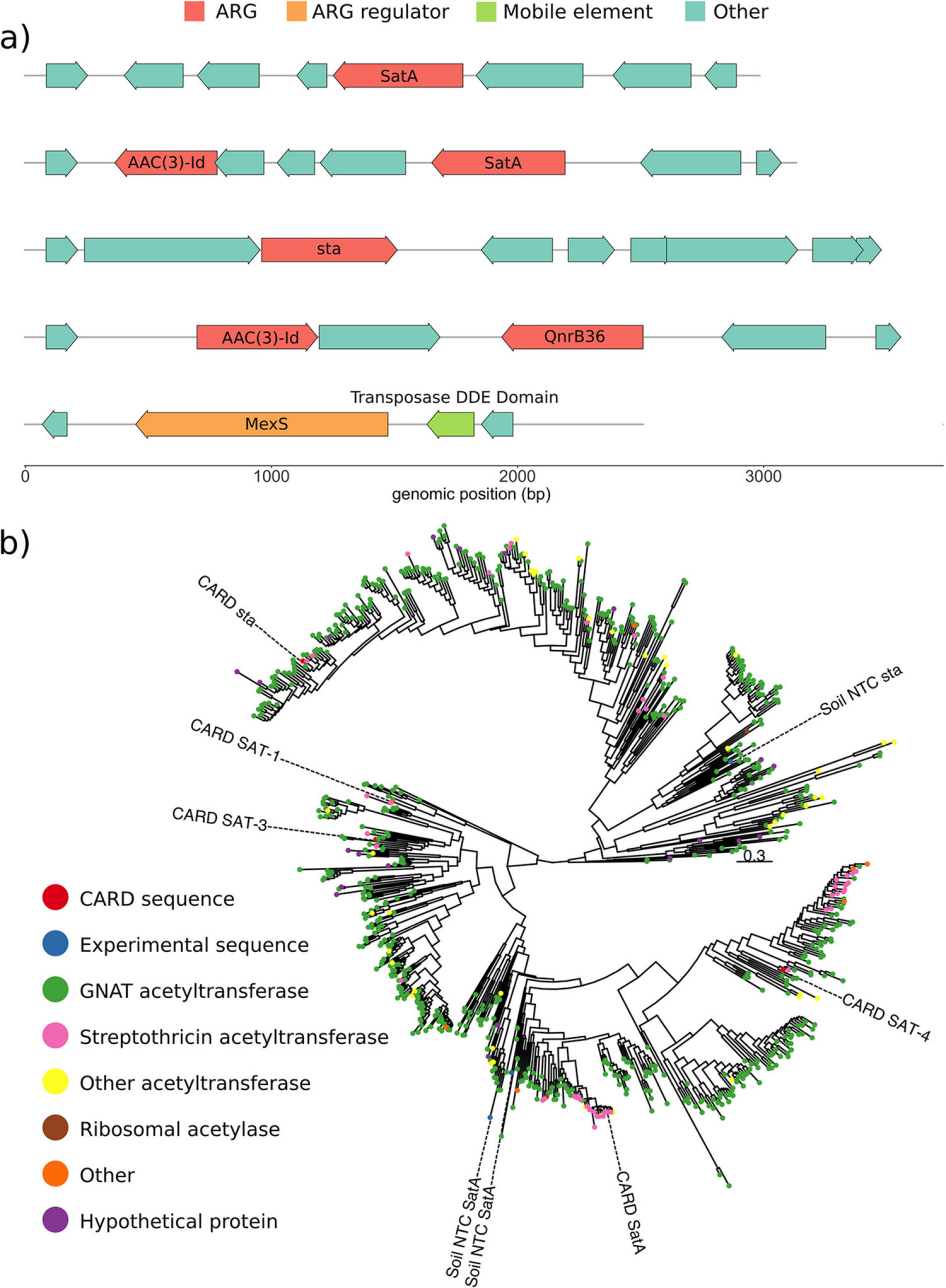
The soil microbiome harbors novel nourseothricin acetyltransferase genes. (a) Genomic context of representative nourseothricin-selected metagenomic inserts (predicted streptothricin acetyltransferases, SatA or sta), including syntenic mobilization or regulatory elements and other antibiotic resistance genes. (b) Phylogenetic tree of five CARD streptothricin acetyltransferase enzymes (red circles; CARD), three soil metagenome nourseothricin resistance genes (blue circles; Soil NTC), and related enzymes.

To demonstrate the utility of the resulting library, we next performed a functional metagenomic selection on the 162-Gb library using the natural product antibiotic nourseothricin. Nourseothricin is a member of the streptothricin class of aminoglycoside antibiotics first described by Waksman and Woodruff in 1942 (53). Following library selection for nourseothricin-resistant colonies, insert amplification (Fig. S2) and sequencing resulted in identification of acetylation to be the dominant mode of resistance in our library (Fig. S4a). Among the putative acetyltransferase enzymes, we identified multiple apparent homologs of known streptothricin acetyltransferases, with some inserts encoding multiple syntenic predicted resistance genes (Fig. 6a). Phylogenetic analysis predicted one soil-derived enzyme to represent a novel cluster distantly related to the streptothricin acetyltransferase StaT enzyme (locus tag soil_nt_15052; 26.22% identity to the closest CARD [Comprehensive Antibiotic Resistance Database] hit) and two other enzymes to cluster with the streptothricin acetyltransferase SatA and Sat-4 enzymes (locus tags soil_nt_08837 and soil_nt_51239; 50.28% and 47.96% identity, respectively, to closest CARD hits) (Fig. 6b; Table S2).

10.1128/mSystems.00524-21.4 TABLE S2 CARD annotations for predicted functionally selected genes. Read ID, locus tag, start and stop positions, functional annotation, antibiotic resistance ontology term, amino acid identity (percent) to CARD sequence, antibiotic resistance drug class, antibiotic resistance mechanism, antibiotic resistance accession number, and experiment ID. Download Table S2, XLSX file, 12.4 MB. Copyright © 2021 Crofts et al. 2021 Crofts et al. https://creativecommons.org/licenses/by/4.0/ This content is distributed under the terms of the Creative Commons Attribution 4.0 International license.

10.1128/mSystems.00524-21.8 FIG S4 Antibiotic resistance gene family abundance. Major gene families predicted by CARD Antibiotic Resistance Ontology (ARO) for (a) soil microbiome selected on nourseothricin (NTC) or goose gut microbiome selected on (b) colistin (CL) or (c) tetracycline (TE). Download FIG S4, JPG file, 0.8 MB. Copyright © 2021 Crofts et al. 2021 Crofts et al. https://creativecommons.org/licenses/by/4.0/ This content is distributed under the terms of the Creative Commons Attribution 4.0 International license.

**FIG 7:**
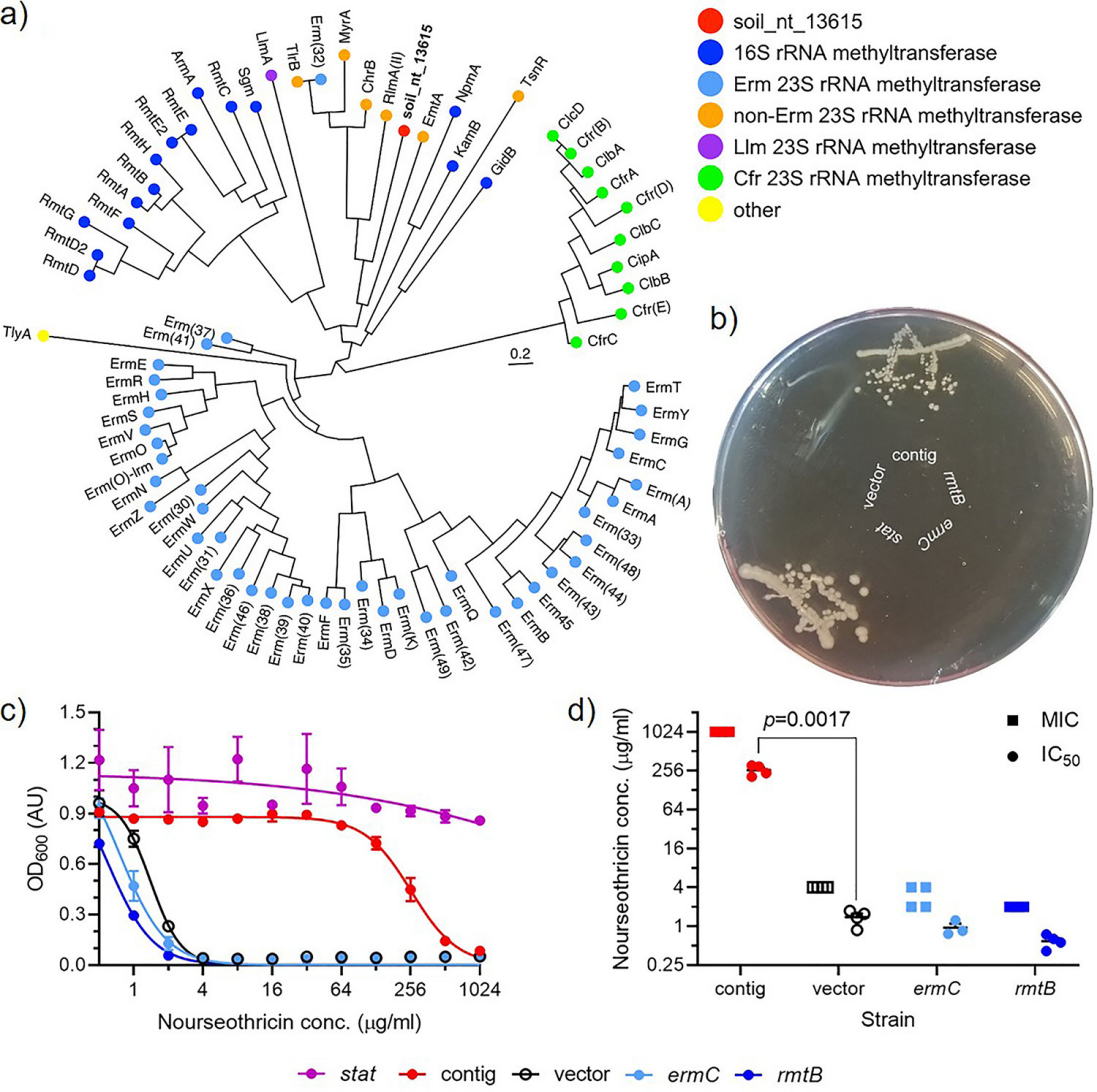
Ribosome methylation may be a new nourseothricin resistance mechanism. (a) Approximate maximum-likelihood phylogenetic tree of CARD rRNA methyltransferase antibiotic resistance genes and the metagenomic predicted methyltransferase (red). (b) Nourseothricin (64 μg/ml) agar plate confirming resistant phenotypes of the subcloned methyltransferase contig (contig)- and streptothricin acetyltransferase (*stat*)-expressing strains and susceptible phenotypes of empty vector control (vector) and representative 16S rRNA and 23S rRNA methyltransferase-expressing strains (*rmtB* and *ermC*, respectively). (c) Nourseothricin broth microdilution assay growth dose-response curves and (d) corresponding MICs and IC_50_s for strains. Error bars represent standard error of 4 replicates, and the *P* value is the result of a heteroscedastic pairwise two-tailed *t* test.

In addition to the novel but recognizable streptothricin acetyltransferase genes, we noted the presence of multiple predicted methyltransferase-encoding genes in the nourseothricin selection (Table S2). This annotation does not fall within the known acetyltransferase-mediated resistance mechanism, and we therefore chose to study the predicted open reading frame soil_nt_13615 (locus tag) found on contig soil_nt_2341 (read ID) to ensure that its presence did not signal an error in our method. Sequence comparison of the predicted amino acid sequence against the CARD database resulted in a 42% identity, 16% coverage hit against MyrA, a 23S rRNA methyltransferase (54), and analysis of the protein by the Conserved Domain Database (55) supported its annotation as an *S*-adenosylmethionine-dependent methyltransferase. Phylogenetic analysis of the putative methyltransferase in the context of all CARD rRNA methyltransferase sequences suggested it to be a novel non-Erm 23S rRNA methyltransferase (Fig. 7a). Subcloning of the entire contig (containing the methyltransferase ORF and a hypothetical protein ORF fragment) into E. coli confirmed its ability to confer resistance to 64 μg/ml nourseothricin, while an empty vector control was susceptible at this antibiotic concentration. For comparison, we also tested three E. coli strains from the Antibiotic Resistance Platform (56); one strain expressing a bona fide streptothricin acetyltransferase gene (*stat*) was resistant, while two strains expressing representative 16S rRNA and 23S rRNA methyltransferase genes (*rmtB* and *ermC*, respectively) showed a susceptible phenotype (Fig. 7b). Broth microdilution assay quantitatively confirmed these findings (Fig. 7c) and demonstrated that expression of the subcloned contig confers a 256-fold increase in nourseothricin MIC over vector control (Fig. 7d).

### METa assembly of functional metagenomic libraries using limited input DNA.

Our experiments comparing METa assembly library creation against library creation using blunt ligation suggest that METa assembly, with its potential for low input DNA mass and higher efficiency, may allow for the creation of functional metagenomic libraries using samples with low DNA mass or quality. We first tested this possibility by creating a mock sample of suboptimal metagenomic DNA consisting of a 10-kb λ phage DNA amplicon (e.g., in place of high-quality DNA >48 kb suggested for use with a Covaris sonicator). We used 200 ng of the DNA as input into a tagmentation reaction (in place of the 2 μg to 20 μg suggested for acoustic fragmentation [37]). Following cleanup, size selection, and repair, we obtained 59.4 ng of 1-kb to 5-kb DNA fragments, all of which were used as input for an assembly reaction. This resulted in a 13.5 ± 1.71-Gb library with an average insert size of 1.02 kb (no empty vectors in 28 colony PCR amplicons) and a library efficiency of 67.55 ± 8.55 Gb/μg of input DNA (Table S1C), suggesting that METa assembly can work well with low-input and lower-quality (i.e., not high-molecular-weight) DNA.

Because it is possible that amplicon DNA behaves differently from metagenomic DNA, we next performed a low-input-mass METa assembly library preparation using the same soil metagenomic DNA as in the 5-μg library. This time, we started with a 250-ng DNA input tagmentation reaction, and after cleanup, size selection, and polymerase repair, we performed an assembly reaction using all 35.2 ng that passed through the processing steps. Electroporation resulted in 3.8 × 10^6^ colonies with an average insert size of 2.08 kb (no empty vectors in 22 colony PCR amplicons). We calculated the library size to be 7.9 ± 0.54 Gb and the library efficiency to be 31.58 ± 2.14 Gb/μg (Table S1C), suggesting that METa assembly can retain high efficiency with low-input metagenomic DNA. Because we had prepared the previous soil library, we did not test this library any further.

Finally, we prepared one additional low-input-mass library. We chose to use metagenomic DNA extracted from a Canada goose fecal pellet in order to include DNA sourced from a nonsoil microbiome. We used 300 ng of input DNA for tagmentation and, following the usual sample processing steps, performed assembly using 60.5 ng of size-selected and repaired inserts. The resulting library was estimated to contain 1.18 × 10^7^ unique clones, with colony PCR indicating that 95.2% of clones contained an insert (1 empty vector in 21 colony PCR amplicons), with an average insert length of 2.39 kb. Together, this suggests a total library size of 27.21 ± 6.15 Gb and a library efficiency of 90.7 ± 20.5 Gb/μg (Table S1C). Notably, across all seven METa assembly libraries constructed in this study, only this single colony out of 153 tested by colony PCR, or 0.65%, was found to be lacking an insert, demonstrating the robustness of the METa assembly approach against library spoilage.

**FIG 8:**
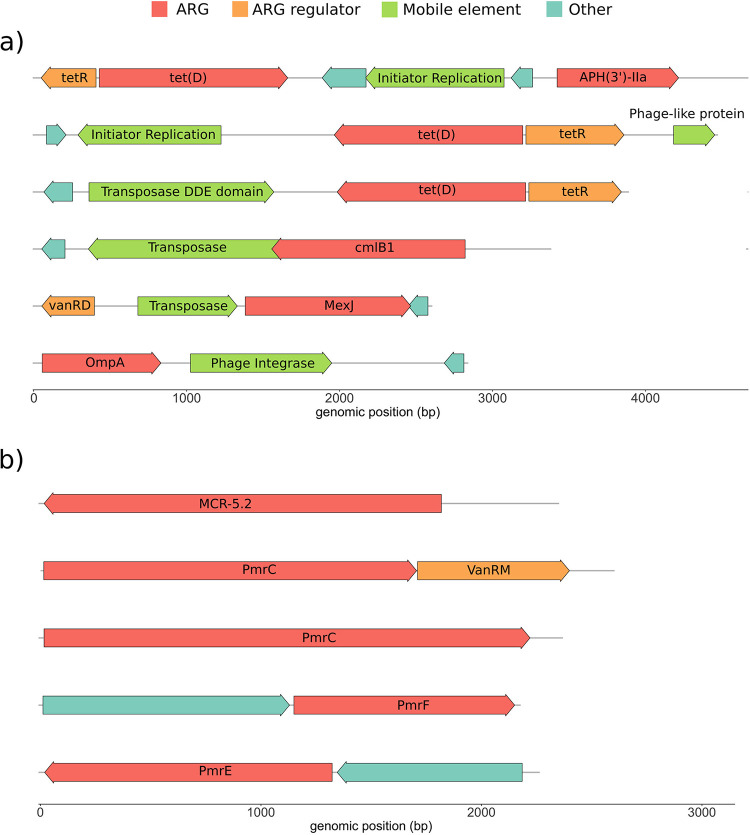
The goose fecal microbiome harbors antibiotic resistance genes against tetracycline and colistin. Genomic context of representative resistance genes, including syntenic mobilization or regulatory elements and other antibiotic resistance genes following goose microbiome library selection on (a) tetracycline or (b) colistin.

We selected the goose fecal microbiome library against a widely used soil natural product antibiotic, tetracycline, and an antibiotic of last resort, colistin. Metagenomic inserts were amplified from functionally selected plasmids by PCR (Fig. S2) and sequenced. The dominant mechanism for tetracycline resistance in the goose gut microbiome appears to be drug efflux (Fig. S4c). Many of the genes encoding efflux pumps were syntenic to known regulatory elements (e.g., *tetR*) and/or potential mobilization elements (e.g., transposase or phage integrase genes) (Fig. 8a). The dominant mechanisms for colistin resistance in our library consist of modification of lipid A and antibiotic efflux (Fig. S4b). Among the predicted lipid A-modifying enzymes, we identified homologs to the emerging MCR family of mobilized colistin resistance enzymes, including a potentially novel MCR-5.2 homolog (locus tag goose_cl_42779) with 36.14% identity to the nearest CARD homolog (Fig. 8b; Table S2).

## DISCUSSION

Functional metagenomic libraries have been valuable tools in microbiome research and have been instrumental in the discovery of novel enzymes involved in antibiotic biosynthesis and resistance, pharmaceutical-microbiome interactions, bioremediation of pollutants, and many other activities of value to medicine and industry (15–17). However, the common use of sonication to fragment DNA necessitates high input DNA mass (e.g., from 2 μg to 20 μg of >48-kb length for a Covaris sonicator), which limits input microbiomes to those with large biomasses. Enzyme-mediated DNA fragmentation can support lower quantities of input DNA, but the inefficiency of blunt-ligation cloning greatly restricts the size of libraries that can be obtained with limited DNA input. These factors have constrained the microbiomes that can be explored by functional metagenomic libraries to those with a large amount of available metagenomic DNA, with upwards of 20 μg of input DNA being recommended for library preparation (37).

Modern sequencing studies use transposases in the Nextera kit to prepare 100-bp to 400-bp DNA fragments from subnanogram inputs (38). We were inspired by this to see if functional metagenomic library preparation could benefit from a tagmentation approach as well. We avoided using commercial Nextera kits because their reagent comes as a mix of preloaded transposomes with different oligonucleotide cargos, which would complicate downstream assembly of inserts into vector. Instead, we used available protocols for the preparation of noncommercial transposase reagent (Fig. S5), which would allow us to specify the oligonucleotide cargo of the resulting transposomes (41, 42).

10.1128/mSystems.00524-21.9 FIG S5 Expression, purification, and testing of transposase activity. (a) Testing auto-induction expression of transposase. (1) Protein ladder with black bars next to 75-kDa and 50-kDa markers; (2) lysate from test expression culture. (b) Transposase purification. (1) Protein ladder with 75-kDa and 50-kDa standards marked; (2) clarified lysate; (3) flowthrough; (4) wash 1; (5) wash 2; (6) 24 h test elution; (7) 48-h elution; (8 to 11) elution washes 1 through 4; (12) ladder. Tn*5*-CDB fusion protein expected mass is ∼75 kDa, and Tn*5* postcleavage expected mass is ∼50 kDa. (c) Testing transposase activity. (1) DNA ladder with 5,000-bp, 1,500-bp, and 500-bp bands marked; (2) DNA treated with full transposase reaction; (3) DNA with transposase reaction minus enzyme; (4 and 5) same as 2 and 3 with column-based kit clean-up; (6) input metagenomic DNA without treatment. Download FIG S5, JPG file, 0.9 MB. Copyright © 2021 Crofts et al. 2021 Crofts et al. https://creativecommons.org/licenses/by/4.0/ This content is distributed under the terms of the Creative Commons Attribution 4.0 International license.

We found that transposase-mediated fragmentation can be controlled to yield DNA fragments roughly the size of bacterial genes (Fig. 3; Fig. S1a) with the following empirical conditions for tagmentation reactions being compatible with our enzyme preparation: 10 mM TAPS buffer (pH 8.5), 5 mM MgCl_2_, 10% (wt/vol) dimethylformamide, input DNA at 10 ng/μl total reaction volume, and loaded transposome at 0.5 ng enzyme per ng of input DNA (i.e., 5 ng of transposome per μl). The resulting inserts are compatible with classic blunt-ligation-based cloning (Fig. 2b) to produce functional metagenomic libraries (Fig. 4). Tagmentation of metagenomic DNA was successful across several DNA sources, including purified bacterial genomic DNA, PCR amplicons, soil metagenomic DNA, and fecal metagenomic DNA.

We realized that installation of mosaic end tags of known sequence on the ends of fragmented DNA could allow us to replace blunt-ligation cloning with a higher-efficiency hybridization-based assembly method (Fig. 2c). We found that libraries prepared using assembly cloning resulted in significantly larger libraries and orders-of-magnitude-higher cloning efficiency than libraries prepared by blunt ligation, demonstrating the dramatic synergy of tagmentation and DNA assembly (Fig. 4; Table S1B). While variation in read counts and the number of input colonies confounded a statistical interpretation, qualitatively it appears that libraries prepared by METa assembly provide equal, if not greater, coverage of input DNA compared to libraries prepared by blunt ligation (Fig. 5a; Fig. S3a).

**FIG 9:**
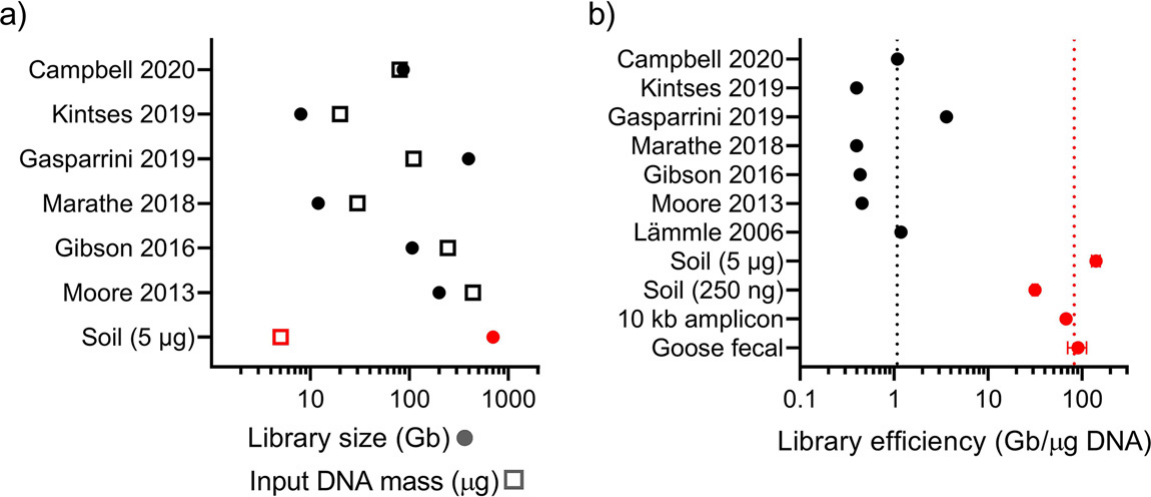
METa assembly libraries use DNA more efficiently than other methods of functional metagenomic library preparation. (a) Input DNA mass and functional metagenomic library sizes for six publications (black) compared to the large (5 μg) soil METa assembly library (red). Error bars (not present for literature libraries) calculated following colony PCR (*n* = 46 successful reactions) represent standard error. Filled circles correspond to library sizes and empty squares correspond to input DNA mass. (b) Library efficiency (library size in Gb normalized to input DNA mass in μg) calculated for seven literature examples (black) or four METa assembly libraries (red). Vertical dotted lines correspond to literature (black) or METa assembly (red) average efficiency (*n* = 7 for literature examples, *n* = 4 for METa assembly examples). Error bars for METa assembly libraries represent standard error and were calculated as before (from top to bottom successful reactions *n* = 46, 22, 28, and 21). Campbell (2020), reference 35; Gasparrini (2019), reference 21; Kintses (2019), reference 51; Marathe (2018), reference 45; Gibson (2016), reference 57; Moore (2013), reference 28; and Lämmle (2006), reference 44.

In order to compare METa assembly against state-of-the-art functional metagenomic library preparation by current experts in the field, we carried out a literature search of recent articles to find appropriate comparators (Fig. S6). Literature-prepared functional metagenomic libraries, with sufficient methods detail to determine library efficiency (library size in gigabases normalized to metagenomic input DNA in micrograms) (21, 28, 35, 45, 51, 57), showed that METa assembly achieves substantial gains in efficiency. Literature protocols used initial DNA input masses of between 20 μg and 440 μg to prepare functional metagenomic libraries sized between 8 Gb and 396 Gb (Table S1C). Starting with 5 μg of metagenomic DNA, we used METa assembly to make a library totaling 703 Gb in size, approximately the same size as the six literature examples combined using a fraction of their total input DNA (Fig. 9a; Table S1C). Similarly, METa assembly can be used to prepare more traditionally sized libraries (i.e., in the tens of gigabases) using only 200 ng to 300 ng of input DNA, less than 10-fold the normative 5-μg input. When library size is normalized to input metagenomic DNA mass to calculate library efficiency, functional metagenomic libraries prepared by METa assembly outstrip libraries prepared by blunt ligation by nearly 80-fold (Fig. 9b).

10.1128/mSystems.00524-21.10 FIG S6 Literature search for functional metagenomic library preparation details. The 125 most recent literature hits for the search terms “functional metagenomics” OR “metagenomic libraries” as well as four articles with detailed functional metagenomic library preparation descriptions were manually searched for sufficient methods detail to determine input metagenomic DNA mass, total library size, and library efficiency. Seven articles had sufficient detail available for further analysis. Download FIG S6, JPG file, 0.2 MB. Copyright © 2021 Crofts et al. 2021 Crofts et al. https://creativecommons.org/licenses/by/4.0/ This content is distributed under the terms of the Creative Commons Attribution 4.0 International license.

We also verified the utility of our libraries as tools for bioprospecting by subjecting them to selection on four classes of antibiotics. We chose the Pseudomonas and *Pandoraea* strains used to prepare our first functional metagenomic library because these previously sequenced strains have been shown to be capable of using the antibiotic penicillin as their sole carbon source (50) via a pathway likely initiated by a β-lactamase enzyme (49). Creation of a functional multigenomic library from these strains would allow us to characterize penicillin resistance genes in a sequence-naive manner and test previous annotations and findings. Interestingly, following selection on agar containing 1 mg/ml penicillin, just 3 of the 11 total predicted ABC07 and ABC10 *bla* genes (48) made up the majority of sequenced contigs (Fig. 5c; Fig. S3c), suggesting that the corresponding β-lactamase enzymes have greater activity.

For our soil library, we chose to select with the streptothricin antibiotic nourseothricin. Surveys of antibiotic-producing bacteria have determined that the streptothricins are among the most common antibiotics in soil ecosystems, with between 10% and 42% of soil actinomycetes potentially being producers (56, 58). Surprisingly for such a widespread antibiotic, there are a limited number of described resistance genes (*sat2-4*, *sttH*, and *nat1*), especially compared to related aminoglycoside antibiotics (59–61). Our selection and subsequent analysis suggest that the streptothricin acetyltransferase family is larger and more diverse than currently thought and likely contains unknown major branches. These branches likely incorporate many enzymes which are now solely annotated as Gcn5-related *N*-acetyltransferases (GNATs) (Fig. 6b). We also uncovered a potentially new mechanism of resistance to streptothricin antibiotics, target modification by rRNA methyltransferase (Fig. 7a). We experimentally verified that expression of a functionally captured predicted methyltransferase-containing contig confers an increase in resistance in E. coli (Fig. 7b), including a 256-fold increase in MIC (Fig. 7c and d). The discovery of these novel (and potentially rare) nourseothricin resistance determinants confirms the utility of very large functional metagenomic libraries which are made possible by the METa assembly method.

For selection of our goose fecal microbiome library, we chose to select on two additional classes of antibiotics (in contrast to aminoglycosides and β-lactams used earlier): tetracycline, due to its widespread historical use in medicine and agriculture, and colistin, due to its importance as an antibiotic of last resort. Migratory birds, including species of goose, likely harbor microbiomes richer in antimicrobial-resistant bacteria than other microbiomes (34, 62). In one study, 50% of migratory birds encoded the emerging colistin resistance gene *mcr-1* within their microbiomes, while the most prevalent antimicrobial resistance genes in these microbiomes are against tetracycline (62). The dominant tetracycline resistance mechanism picked up by our experiment was drug efflux (Fig. S4c). As illustrated in the representative insert gene maps for this selection (Fig. 8a), many tetracycline efflux pumps from the goose fecal microbiome are syntenic to predicted mobilization elements, including transposases, and phage- and plasmid-associated genes. In contrast, we found the dominant colistin resistance mechanism to be lipid A modification (Fig. S4b), and our functional metagenomic library selections identified a potentially novel MCR enzyme homolog (Fig. 8b), further confirming the presence of these concerning genes in migratory birds.

In summary, our experiments developing and testing METa assembly highlight several advantages of the method. First, the use of transposases to fragment metagenomic DNA has several benefits. It removes the need for costly capital equipment, such as acoustic fragmentation instruments, while providing the benefits of greater control and experimental flexibility seen with restriction enzyme-mediated fragmentation without the confounders of restriction site frequency or DNA methylation (63). Like sonication, tagmentation shows very little or no sequence bias, making it essentially random (38, 40), but unlike sonication, it can be applied to low-biomass samples. The compatibility with low-biomass samples is especially useful, as low-biomass or rare microbiomes force researchers to increase input DNA mass by pooling independent samples (21, 35, 64) or using potentially biased DNA amplification techniques (45, 65, 66). One particularly relevant low-biomass sample for METa assembly could be clinical swabs, which have been found on average to yield 371 ng of metagenomic DNA (67), which is compatible with the inputs we used here (200 ng to 300 ng).

Second, the addition of mosaic end sequences to the DNA inserts allows the use of modern assembly cloning methods. Random fragmentation of metagenomic DNA by sonication or enzymatic digestion (by restriction enzymes [44] or fragmentases [45]) results in DNA fragments with little or no information about the DNA sequence at the fragment ends. These fragments are limited to lower-efficiency blunt-ligation cloning as opposed to more efficient assembly cloning methods that rely on insert-vector hybridization over ca. 20 bp to drive ligation specificity and efficiency. Assembly cloning and transposase fragmentation are therefore synergistic: without assembly cloning, tagmented DNA would be cloned via blunt ligation with no gains in efficiency, and without tagmentation, assembly cloning fails for lack of insert-vector hybridization. Furthermore, we used a simple inverse PCR step to incorporate mosaic end sequences into our plasmid of choice, demonstrating that most functional metagenomic library plasmids could be adapted to take advantage of the METa assembly workflow as well.

Third, plasmids used in blunt cloning often undergo phosphatase treatment to decrease the chances of self-ligation reactions, which result in clones with empty colonies. Assembly cloning does not face this issue, because exonucleases and a higher incubation temperature prevent nonspecific ligation reactions. We confirmed that phosphatase treatment was not necessary for successful assembly cloning (Table S1B), and our omission of phosphatase treatment allowed us to test the hypothesis that METa assembly is more robust to library spoilage (i.e., formation of empty vectors or incorporation of contaminating DNA). In our hands, blunt ligation in the absence of phosphatase treatment resulted in 31% to 50% of clones containing an empty vector (Table S1B; Fig. S1b). This number is somewhat high compared to other functional metagenomic libraries prepared with phosphatase treatment, ranging from 2% to 34% of clones (44, 68, 69), but is dramatically higher than the 0.65% of METa assembly clones, demonstrating the robustness of assembly. Artificially increasing the proportion of blunt-ligation colonies with an insert to 1.0 would not significantly close the gap in library size or efficiency with the METa assembly libraries.

Finally, the METa assembly protocol presents significant time savings. The process of fragmenting DNA by tagmentation takes only 7 min, followed by a 5-min quench. In contrast, fragmentation by sonication with, e.g., a Covaris E220 instrument requires a 60-min degassing time on top of 20 min or more of fragmentation time. End repair with DNA polymerase to fill in 3′ overhangs following tagmentation takes 15 min (though it could likely be accomplished in less time), while end repair following physical fragmentation of DNA with an End-It kit requires 45-min reactions and a 10-min heat inactivation. Most notably, assembly-based cloning takes 15 min, while it is generally recommended that blunt-ended ligation reaction mixtures be allowed to incubate overnight for optimal efficiency. It has been suggested that functional metagenomic library preparation could be used in a rapid workflow for clinical detection of resistance genes (25). The time savings found in METa assembly, most notably in the cloning step, could be invaluable in such a workflow.

In conclusion, the synergistic combination of fragmentation by transposase and cloning by assembly allows METa assembly to prepare larger, less DNA-greedy, and more robust (i.e., resistant to spoilage by contamination and empty vectors) functional metagenomic libraries. The advantages of METa assembly of functional metagenomic libraries could allow these valuable tools to be prepared from sources previously out of reach, including those of low biomass (such as from the built environment), requiring fast turnaround (such as in the clinic), or of limited availability (such as exotic or historical samples).

## MATERIALS AND METHODS

### Tagmentation of high-molecular-weight DNA to produce 1-kb to 10-kb fragments.

High-molecular-weight DNA for use as a test substrate was obtained from cultures of Pseudomonas sp. strain PE-S1G-1 (referred to as ABC07) and *Pandoraea* sp. strain PE-S2T-3 (referred to as ABC10) (48–50). Each strain was grown in 1 ml of LB supplemented with 100 μg/ml carbenicillin (LB+CA100) at 30°C for 48 h, and genomic DNA was extracted using a Quick-DNA high-molecular-weight kit (Zymo Research, catalog no. D6060) according to the manufacturer’s protocol, quantified, and combined to give a 100-ng/μl stock solution with equal input by mass from each genome. Quantification of DNA for all experiments, unless otherwise noted, was performed using a QuantiFluor One double-stranded DNA (dsDNA) system (Promega, catalog no. E4871).

Tagmentation reaction mixtures were prepared in 20-μl volumes in 1× TAPS-DMF buffer {10 mM [tris(hydroxymethyl)methylamino]propanesulfonic acid [TAPS] buffer [pH 8.5], 5 mM MgCl_2_, and 10% [vol/vol] dimethylformamide} containing 200 ng of DNA and transposome in concentrations ranging from 0 ng enzyme per ng DNA to 2 ng enzyme per ng DNA. Following incubation for 7 min at 55°C, the reactions were quenched by addition of sodium dodecyl sulfate (SDS) to a final concentration of 0.05%, and the mixtures were further incubated for 5 min at 55°C. Next, 6× loading dye was added to each reaction mixture, and samples were analyzed by pulsed-field gel electrophoresis at 4°C using a Pippin Pulse power supply (Sage Science, catalog no. PPI0200) running preset protocol no. 4: 16 h at 75 V on a 0.75% Tris-acetate-EDTA (TAE) agarose gel. Following electrophoresis, the gel was stained with SYBRSafe and visualized under UV light.

### Preparation of pZE21-ME vector.

The pZE21 plasmid (70) was used as a template for an inverse PCR to replace the multiple cloning site with tandem mosaic end sequences. The resulting vector (pZE21-ME) contains the following sequence, with linearization occurring at the slash (/): 5′-AGATGTGTATAAGAGACAG/CTGTCTCTTATACACATCT-3′. The construct was amplified by 2-step PCR according to manufacturer recommendations using Q5 high fidelity polymerase (New England Biolabs, catalog no. M0494S) with primers 6469TSC and 6470TSC (Table S1A). The vector product was digested with DpnI to remove pZE21 vector, sized, purified from an agarose gel, and circularized by using an End-It DNA End-Repair Kit (Lucigen, catalog no. ER0720) and a Fast-Link DNA Ligation Kit (Lucigen, catalog no. LK0750H) following manufacturer instructions. Unless otherwise noted, agarose gel experiments were conducted using ∼0.7% agarose precast with SYBRSafe (Invitrogen catalog no. S33102) following the manufacturer’s recommendations. E. coli DH10B (New England Biolabs 10-beta, catalog no. C3020K) was transformed with the construct, selected on LB supplemented with 50 μg/ml kanamycin (LB+KAN50), and frozen at −80°C as a 15% glycerol stock. Incorporation of the mosaic end sequences 23 bp downstream of the pZE21 ribosome binding site, analogous to the often-used pZE21 HincII restriction site, was confirmed by Sanger sequencing. When used as a vector in cloning reactions, pZE21-ME was prepared as described above (inverse PCR followed by DpnI digestion and gel purification). Phosphatase treatment to remove 5′ phosphate groups was omitted because vector linearization by Q5 polymerase inverse PCR results in unphosphorylated amplicons, while high temperatures and 5′ exonucleases used in downstream assembly reactions prevent almost all vector self-annealing.

### Comparison of METa assembly to blunt ligation for functional metagenomic library preparation.

To compare the efficiency of METa assembly against blunt-ligation cloning, we prepared triplicate functional multigenomic libraries using as input the mixed genomic DNA from Pseudomonas sp. strain PE-S1G-1 (ABC07) and *Pandoraea* sp. strain PE-S2T-3 (ABC10). A 1-ml bulk tagmentation reaction mixture consisting of 1× TAPS-DMF buffer, 10 μg mixed genomic DNA (10 ng input DNA per μl [final volume]), and 5 μg transposome (0.5 ng of transposome per ng of input DNA) was prepared and was incubated and quenched as described above. The bulk reaction was purified and concentrated using a silica column-based kit (New England Biolabs, catalog no. T1030S) according to the manufacturer’s directions for targeting fragments >2 kb in length. Tracking dye was added to the elution mixture, and the entire sample was loaded onto an agarose gel and run at 70 V for 120 min alongside a λ DNA BstEII digestion ladder (New England Biolabs, catalog no. N3014S). Prior to running, the electrophoresis apparatus and gel tray were washed with Milli-Q water, soaked in 10% bleach for 15 min, and rinsed with Milli-Q water. The bleaching process and use of λ DNA ladder are both necessary to prevent contamination of the metagenomic DNA fragments by foreign DNA that can be mistakenly incorporated during blunt-ligation cloning (37). Fragments between ∼1 kb and ∼8 kb were size selected by excision with a clean razor blade, and DNA was purified from the agarose by a silica column-based gel extraction kit (New England Biolabs, catalog no. T1020S; used in all gel extraction steps) with ∼500 mg of agarose used per purification column. For downstream cloning reaction calculations, the average insert size was taken to be ∼2.2 kb. Triplicate METa assembly and blunt-ligation reactions both used this pool of inserts as their input DNA.

For triplicate METa assembly reactions, DNA inserts were first subjected to an end-filling step. Triplicate end-filling reactions were performed by combining 7 μl of Q5 high-fidelity polymerase previously heated to 98°C for 30 s with 7 μl of DNA fragments (300 ng each) and incubated at 72°C for 15 min to fill in 5′ overhangs. DNA fragments were purified by silica column as before. Triplicate assembly reactions were prepared using NEBuilder HiFi DNA assembly enzyme mix according to manufacturer’s protocols containing an ∼2:1 ratio of insert to vector as follows: 10 μl of 2× NEBuilder HiFi master mix (New England Biolabs, catalog no. E2621S), 40 ng of DNA fragments (0.03 pmol), 20 ng pZE21-ME (0.015 pmol), and Milli-Q water to a volume of 20 μl. Reaction mixtures were incubated at 50°C for 15 min and then transferred to ice. Triplicate sham reactions using mixtures containing Milli-Q water in place of inserts were performed in parallel.

Triplicate blunt-ligation reactions were prepared to follow established functional metagenomic library cloning protocols (21, 37). DNA fragments were blunted using an End-It DNA end repair kit (Lucigen, catalog no. ER0720) according to the manufacturer’s protocol, with triplicate 50-μl reaction mixture containing 300 ng of DNA fragments, 5 μl of 10× end repair buffer, 5 μl of deoxynucleoside triphosphate (dNTP) mix, 5 μl of ATP mix, and 1 μl of end repair enzyme mix in Milli-Q water. The reaction mixture was held at room temperature (ca. 23°C) for 45 min, heat inactivated at 70°C for 10 min, and purified by silica column. Triplicate blunt-ligation reaction mixtures were prepared with an ∼5:1 insert-to-vector ratio, with each 15-μl reaction mixture containing 100 ng of end-repaired DNA (0.075 pmol), 20 ng of pZE21-ME (0.015 pmol), 1.5 μl of Fast-Link 10× ligation buffer, 0.75 μl ATP solution (10 mM), and 1 μl of Fast-Link DNA ligase (Lucigen, catalog no. LK0750H). Blunt-ligation reaction mixtures were incubated at room temperature (ca. 23°C) overnight and then heat inactivated at 70°C for 15 min. Triplicate sham reactions using mixtures containing all the above components except insert were performed as well.

Products of all 12 reactions (two techniques with triplicate insert reactions and triplicate sham reactions each) were purified and desalted with a silica column kit. For each reaction, the entire 10-μl Milli-Q water elution was added to a 25-μl aliquot of commercial 10-beta electrocompetent E. coli DH10B cells (advertised transformation efficiency of >2 × 10^10^ CFU/μg DNA) in a 0.1-cm Gene Pulser cuvette (Bio-Rad, catalog no. 1652083) and electroporated on an Electroporator 2510 instrument (Eppendorf) at 1.8 kV with default settings of 10-μF capacitance and 600-Ω resistance (note that this results in a τ constant similar to that seen with standard settings on other instruments of 25-μF capacitance and 200-Ω resistance). Cells were immediately rescued in 1 ml of 37°C SOC outgrowth medium (Super Optimal Broth with Catabolite repression; New England Biolabs, catalog no. B9020S) and incubated with shaking at 37°C for 1 h. Following recovery, 100 μl of 100-fold (10^2^)-, 10,000-fold (10^4^)-, and 1,000,000-fold (10^6^)-diluted cultures were plated onto LB+KAN50 plates overnight at 37°C. The remaining stocks of the sham reactions were discarded, while the remaining stocks of the insert reactions were individually used to inoculate 50 ml of LB+KAN50 and shaken overnight at 18°C before being transferred to a 37°C shaking incubator the next day to amplify the libraries to an optical density at 600 nm (OD_600_) of between 0.6 and 1.0 AU. The amplified libraries were concentrated by centrifugation at a relative centrifugal force (rcf) of 4,000 × *g* for 7 min and resuspended to 10 ml in 15% glycerol in LB+KAN50. The concentrated libraries were aliquoted 1 ml at a time into cryovials for storage at −80°C.

To estimate average insert size and the rate of successful insert capture, colony PCR was performed for 15 colonies per library and 1 colony per sham library. Each 12.5-μl reaction mixture contained 0.25 μl each of 10 μM primers 6463TSC and 6464TSC (Table S1A), 6.25 μl of OneTaq quick-load 2× master mix (New England Biolabs, catalog no. M0486), and Milli-Q water to 12.5 μl. The reaction mixtures were incubated in a thermocycler with the following program: 5 min at 94°C followed by 25 cycles of 30 s at 94°C, 45 s at 62°C, and 8 min at 68°C, followed by 5 min at 68°C and storage at 4°C. Subsequent colony PCR amplifications were performed using Q5 polymerase according to manufacturer’s recommendation with the following quicker 2-step thermocycler program: 98°C for 3 min, 25 cycles of 98°C for 10 s and 72°C for 4 min, followed by holding at 72°C for 5 min. Reaction products were analyzed on agarose gels, and average insert size was calculated by comparison to a DNA ladder as follows. The migration distance of each band of the DNA ladder and that band’s corresponding log_10_(length in kilobases) were plotted and fitted to a line. This linear equation, unique to each gel, was used to convert migration distance of colony PCR product into amplicon lengths. Because the colony PCR primers amplify 500 bp of vector backbone in addition to the full length of each insert, this length (500 bp) was subtracted from each calculated amplicon to give an accurate estimated insert size. The proportion of colonies with an insert was estimated by taking the proportion of reactions returning a >500-bp product (500 bp being the expected amplicon size for a vector backbone-only reaction) over the total number of successful reactions. The library size for each assembly or cloning reaction was calculated using the following formula (where CFU is calculated as CFU/ml × 1 ml total recovery volume): (CFU × proportion of colonies with inserts × average insert size [in base pairs])/(10^9^ bp/Gb).

Standard errors for library size, incorporating standard error of insert size and standard error of empty vector proportion, were calculated using the Dantas lab Library Size Calculator site: http://dantaslab.wustl.edu/LibSizeCalc/. Following library size estimation, each library was normalized to the quantity of insert DNA used for the cloning step (i.e., 40 ng for METa assembly and 100 ng for blunt ligation) for direct comparison across cloning techniques in the form gigabases of library/nanograms of insert DNA (cloning efficiency).

### Assembly of a large soil microbiome metagenomic library by METa assembly.

Soil metagenomic DNA (Text S1) was used as input for a 5-μg tagmentation reaction performed in 1× TAPS-DMF buffer with a 10-ng/μl final metagenomic DNA concentration and 0.5 ng of transposome per ng of metagenomic DNA. The reaction mixture was incubated at 55°C for 7 min and then quenched for 5 min at 55°C by adding SDS to 0.05%. The reaction was purified and concentrated by silica column kit and eluted twice with 6 μl of 55°C elution buffer. To the full elution was added 1 μl of 6× tracking dye and 2 μl of glycerol before loading onto an agarose gel and running at 75 V for 2 h alongside a λ BstEII digest ladder. Fragments were size selected by excision from the agarose gel, targeting inserts between 1 kb and 6 kb in length, purified by silica column, and eluted twice with 25 μl elution buffer heated to 55°C. Fragment overhangs were filled by PCR with 50 μl of Q5 2× master mix for 15 min at 72°C, purified with a silica column, eluted twice with 6 μl of 55°C water, and quantified. From a total of 1.122 μg of available purified inserts, two libraries were assembled. The first test library used 175 ng of inserts (∼0.14 pmol assuming a 2-kb average size) assembled into 100 ng of pZE21-ME (∼0.07 pmol) for a 2:1 insert-to-vector ratio using NEBuilder HiFi enzyme mix as before in a 20-μl total volume. The second library used the remaining 887 ng (0.718 pmol) of inserts (the unaccounted-for 60 ng was likely lost in pipetting) in a scaled-up reaction with 505.8 ng (0.359 pmol) vector and 53.85 μl of NEBuilder HiFi master mix in a total volume of 107.7 μl, again maintaining a 2:1 insert-to-vector ratio. The reaction was scaled in order to keep the total quantity of DNA fragments within the manufacturer’s recommended 0.03 to 0.2 pmol per 20 μl of reaction mixture.

The assembly reaction products were desalted by silica column purification, eluted twice with 6 μl 55°C water (the 175-ng assembly) or twice with 25 μl 55°C water (the 887-ng assembly), and electroporated into 25 μl (the 175-ng assembly) or 125 μl (the 887-ng assembly) of NEB 10-beta electrocompetent E. coli cells at 1.8 kV. The libraries were amplified overnight in LB+KAN50, concentrated, and stored in 1 ml 15% glycerol stocks as before. Library sizes were quantified by colony counting and colony PCR as before. Library efficiency, distinct from the previously calculated cloning efficiency, was calculated by normalizing the final total library size to the mass of input metagenomic DNA (as opposed to normalization to inserts used at the cloning step for cloning efficiency).

### METa assembly of soil or goose fecal metagenomic libraries using limited input DNA.

A library to test the limits of METa assembly was prepared using a 10-kb DNA amplicon as input. To generate the input DNA for this assembly, a 50-μl Q5 PCR using template DNA and primers from a Phusion HiFi amplification control (Thermo Scientific, catalog no. F553S) was run as follows: 25 μl of Q5 2× hot start master mix, 6.25 μl of 4 μM primers P1 and P2, 1 μl of template DNA (Thermo Scientific, catalog no. F553S), and Milli-Q water up to 50 μl. Amplification was carried out according to manufacturer’s protocols with an annealing temperature of 65°C. The size and purity of the 10-kb product were verified by agarose gel electrophoresis, and the product was purified with a silica column and used as the substrate in a 200-ng, 20-μl tagmentation reaction (10 ng DNA per μl reaction mixture, 0.5 ng transposome per ng input DNA). Size selection, overhang filling, and purification were performed as before with inserts assumed to have an average length of 2.2 kb. Fragments (59.4 ng, 0.044 pmol) were assembled into pZE21-ME vector (30 ng, 0.022 pmol) at a 2:1 molar ratio with NEBuilder HiFi assembly master mix (total volume, 20 μl) and purified, and 10-beta E. coli cells were transformed to produce a library for quantitation by colony counting and colony PCR. Library efficiency (gigabases per microgram of DNA used in the tagmentation step) was calculated as before.

A soil functional metagenomic library was prepared as described above, with 250 ng of previously extracted soil metagenomic DNA used as input in a 25-μl tagmentation reaction. The reaction product was purified with silica columns, and fragments between 2 kb and 6 kb in length were purified by extraction from agarose gels. Overhang filling, size selection, and assembly by NEBuilder HiFi master mix with an estimated ∼2-fold excess of inserts (35.2 ng, 0.029 pmol) to vector (21 ng, 0.015 pmol) in a 20-μl total volume were performed as before, followed by assembly purification and electroporation into 10-beta E. coli. The library was quantified by colony counting and colony PCR, amplified overnight as described above, and stored at −80°C in 10 1-ml aliquots in 15% glycerol in LB+KAN50. Library efficiency (gigabases per microgram of metagenomic DNA) was calculated as before.

To prepare a goose fecal microbiome library, a freshly voided adult Canada goose (*Branta canadensis*) fecal pellet was collected from the Northwestern University campus (coordinates: 42.056084, −87.670661). Fecal pellet collection was approved by the Northwestern University Institutional Animal Care and Use Committee (IACUC) under protocol EC20-0252. Within 15 min of collection, 474 mg of fecal pellet was used as input for metagenomic DNA extraction using a DNeasy PowerSoil kit (Qiagen, catalog no. 12888-100) following modifications for goose microbiome extraction suggested by Cao et al. (62). Briefly, these consisted of incubating the sample suspended in Qiagen buffer CD1 at 65°C for 10 min followed by incubation at 95°C for 10 min followed by the kit manufacturer’s protocol.

Tagmentation was performed as described above (10 ng input DNA per μl volume, 0.5 ng of loaded transposome per ng of input DNA) using 300 ng of input DNA. The tagmentation reaction was quenched, purified, and loaded onto an agarose gel as before. DNA fragments between ca. 1.7 kb and 6.3 kb were collected and purified by gel extraction as described above, eluting twice with 6 μl of 55°C Milli-Q water. To the 12-μl elution mixture was added 12 μl of 2× Q5 master mix previously held at 98°C for 30 s, and the gap-filling reaction mixture was held at 72°C for 15 min before purification and elution, resulting in 60.5 ng of blunt-ended DNA fragments. All 60.5 ng of insert DNA (∼0.05 pmol assuming a 2-kb average size) was added to a 20-μl NEBuilder HiFi assembly reaction mixture with 35 ng of linear pZE21-ME vector (∼0.025 pmol, 2:1 insert-to-vector ratio) and held at 50°C for 15 min, followed by column purification and elution twice with 6 μl of 55°C Milli-Q water. The purified assembly reaction was electroporated into an aliquot of electrocompetent E. coli 10-beta cells, rescued, and amplified; CFU counts were taken, and colonies were used as input for PCR to determine average insert length and library size as described above. Library efficiency (library gigabases/microgram of input DNA) was calculator as before.

### Extraction of plasmid DNA from unselected libraries.

Functional metagenomic library stocks prepared from strains ABC07 and ABC10 (see above) were plated in triplicate on LB+KAN50 agar to determine concentrations (CFU per milliliter). Each triplicate library was plated again on LB+KAN50 agar plates with volumes calculated to result in ∼1,000 colonies being plated, resulting in an average of ∼600 colonies per plate following overnight incubation at 37°C. Colonies on each plate were collected by addition of 750 μl of LB to the plate, resuspension of colonies using a cell spreader, and removal of the medium. This process was repeated, resulting in ∼1 ml of recovered bacterial suspension, which was subsequently used as input for plasmid purification with a miniprep kit (New England Biolabs, catalog no. T1010S). The pooled plasmid library DNA for each library was eluted in 35 μl of elution buffer and quantified, resulting in an average concentration of 58 ng/μl miniprep.

### Functional metagenomic selection for antibiotic resistance.

Libraries to be tested were first plated on LB+KAN50 agar plates to determine the titer (CFU per milliliter), following which the volume of frozen stock necessary to provide 10-fold coverage of unique inserts in each library was calculated. This volume, brought up to 100 μl with LB medium if necessary, was plated on Mueller-Hinton II cation-adjusted agar plates (BD BBL, catalog no. 212322) containing 50 μg/ml kanamycin (MH+KAN50) and another selective antibiotic depending on the library. Triplicate ABC07/ABC10 libraries prepared by METa assembly or blunt ligation were plated on MH+KAN50 supplemented with 1,000 μg/ml (1 mg/ml) penicillin G sodium salt (Fisher Scientific, catalog no. AAJ6303214). The “175-ng assembly” soil library (162 Gb) was plated on MH+KAN50 supplemented with 64 μg/ml nourseothricin sulfate (Dot Scientific, catalog no. DSN51200-1), and the 300-ng goose fecal pellet library was plated on MH+KAN50 supplemented with either 8 μg/ml tetracycline (Fisher Scientific, catalog no. AAJ6171406) or 4 μg/ml colistin sulfate (Fisher Scientific, catalog no. AAJ6091503). Following overnight selection of plates at 37°C, resistant colonies were collected as slurries, and plasmids were extracted and purified as described above. Antibiotic concentrations were chosen based on literature precedent (i.e., 1 mg/ml penicillin [49, 50], 8 μg/ml tetracycline [37], 4 μg/ml colistin [35], and 64 μg/ml nourseothricin [71]). Complete growth inhibition of E. coli DH10B with empty pZE21-ME vector was confirmed for each antibiotic after plating a similar high-density lawn and incubating at 37°C overnight.

### Amplicon sequencing of functional metagenomic libraries.

Plasmid minipreps from unselected and antibiotic selected libraries were used as templates for PCRs targeting vector inserts. Seven 100-μl PCRs, one for each library, were performed, each using 50 μl of Q5 2× master mix, 39 μl water, 5 μl each of primers 6463TSC and 6464TSC at 10 μM (Table S1A), and 1 μl of library miniprep corresponding to between 5.4 ng and 8.6 ng of DNA. Reactions were run in a thermocycler using the following settings: holding at 98°C for 30 s, followed by 16 cycles of 98°C for 10 s and 72°C for 4 min, followed by holding at 72°C for 5 min. Reaction products were purified with silica columns, and each column was eluted twice with 20 μl of 55°C water and quantified. Insert amplicon integrity was verified by running 100 ng of purified DNA from each reaction on an agarose gel.

Purified amplicons were shipped overnight to the University of Illinois at Urbana-Champaign Roy J. Carver Biotechnology Center as 30-μl aliquots containing 500 ng of each reaction mixture. Samples were used as input for library preparation and sequencing on the PacBio Sequel II platform at the center as follows. Amplicons were ligated to barcoded adaptors using a barcoded overhang adapter kit (Pacific Biosciences, CA). The barcoded amplicons were normalized to the estimated number of unique inserts, based on colony counts, and pooled. The pooled amplicons were used as input for a SMRTbell Express template prep kit 2.0 (Pacific Biosciences) to prepare the sequencing library. The library was quantitated by Qubit fluorometer, and DNA fragment size and quality were confirmed on a fragment analyzer (Agilent, CA). The library was sequenced on a SMRT Cell 8M on a PacBio Sequel II instrument with a 20-h movie time. Circular consensus analysis was performed on the resulting BAM file using SMRTLink V8.0 with the following parameters: ccs –min-length 500 –max-length 12000 –min-passes 3 –min-rq 0.99. Demultiplexing was performed with lima (Pacific Biosciences) using default parameters.

### Analysis of functional metagenomic library sequencing.

Read coverage and evenness of the ABC07/ABC10 libraries were determined for each genome individually. Long reads were mapped to either ABC07 (ASM217990v1) or ABC10 (ASM217996v1) assemblies using minimap2 v2.17-p94 with default settings, converting resulting SAM files to BAM files with SAMtools v1.9 (72) using default settings, and calculating the average read coverage over a 1,000-bp window using pileup.sh from the BBmap v38.86 suite (https://jgi.doe.gov/data-and-tools/bbtools/). For visualization of coverage, a rolling average was produced using the rollmean() function from the zoo R package v1.8.8 (73).

To identify potential β-lactamase genes in the ABC07 (ASM217990v1) and ABC10 (ASM217996v1) genomes, the amino acid FASTA files were downloaded and individually submitted to the Comprehensive Antibiotic Resistance Database (CARD) Resistance Gene Identifier (RGI) web server (https://card.mcmaster.ca/analyze/rgi) (54) for prediction of all resistance genes using the parameters “Protein sequence,” “Perfect, Strict and Loose,” “Include nudge,” and ‘High quality/coverage.” The resulting data frame was filtered for antibiotic resistance genes belonging to the β-lactamase AMR gene family that had “antibiotic inactivation” as its resistance mechanism to generate a list of ABC07- or ABC10-specific β-lactamase amino acid sequences. For each functional metagenomic approach and selection method, the following was performed on all reads that mapped to either ABC07 or ABC10. Open reading frames (ORFs) were identified using Prokka v1.14.6 (74) with the parameters –norrna, –notrna, –noanno, and –fast. Resulting ORFs for each combination were clustered with mmseqs2 linclust (75) with the parameters –min-seq-id 0.95 and -c 0.95. Predicted β-lactamases were searched against the set of representative ORFs using an E value cutoff of 10^−6^. Afterwards, a single best hit determined by bit score, identity, and coverage was kept. A hit was considered a true β-lactamase if it had ≥95% coverage and identity.

Functional annotation of the soil and goose fecal microbiome libraries was carried out by ORF searching and clustering all metagenomic long reads using Prokka and mmseqs2 linclust as previously described. Representative ORFs were submitted to the CARD RGI portal to search for antibiotic resistance genes using the previously stated parameters. Nonresistance functional annotations were obtained by submitting representative ORFs to eggNOG-mapper v2 (http://eggnog-mapper.embl.de/) (76). Antibiotic resistance genes and functional annotations were then mapped back to all identified ORFs using a custom script. Counts of antibiotic resistance genes, antibiotic resistance gene ontology, superfamilies, and drug class were extracted from the CARD RGI output.

Mobile genetic elements syntenic to antibiotic resistance genes were identified based on keyword searches of the eggNOG “annotation” column using the search terms “transposase,” “conjugative,” “phage,” “integrase,” “replication,” and “recombinase.” ORFs that matched these search terms were further verified using UniProt (https://www.uniprot.org/) (77). Plots were made using the R package ggplot v3.3.2 (78), and gene region visualizations were created using the R package gggenes v0.4.0.

Streptothricin acetyltransferase gene family phylogenetic analysis was performed by extracting the five amino acid sequences for streptothricin resistance enzymes from the CARD database and using these as input to run NCBI BLASTp (79) against the NR database (run November 2020). Sequence hits were filtered for ≥25% amino acid identity and ≥70% alignment length to match CARD workflow. Replicates were removed from the combined sequences followed by clustering using CD-Hit (80) at a 90% identity threshold. The amino acid sequences of three representative putative streptothricin acetyltransferase enzymes from the nourseothricin selection were added to the sequence file, and all were aligned using MAFFT v7.471 (81) with the parameters –thread 8 –localpair –maxiterate 1000. An approximate maximum-likelihood phylogenetic tree was calculated using FastTree v2.1.11 (82) with the parameter -wag. The resulting phylogenetic tree was visualized with ggtree (83).

Antibiotic resistance conferring ribosomal methyltransferase phylogenetic analysis was performed as with the acetyltransferase analysis. rRNA methyltransferase protein sequences were downloaded from CARD. These sequences and the predicted amino acid sequence of the metagenomic methyltransferase were aligned and used to generate and visualize an approximate maximum-likelihood phylogenetic tree using the parameters described above.

### Subcloning and testing of a putative methyltransferase contig from nourseothricin selection.

PacBio sequences arising from the 64-μg/ml nourseothricin selection were examined for potentially missed resistance mechanisms. One insert appearing on several contigs that contained a predicted methyltransferase gene and the beginning of a gene encoding a hypothetical protein was selected for functional validation. Primers 6562TSC and 6563TSC (Table S1A) were used to amplify the metagenomic insert using the nourseothricin selection miniprep as the template. The resulting amplicon was digested with DpnI for 1 h to remove other plasmids and gel purified. The purified amplicon was cloned into pZE21-ME vector in a scaled-down 5-μl NEBuilder HiFi assembly reaction, and 2 μl of the reaction mixture was electroporated directly into 10-beta E. coli electrocompetent cells. Following recovery in 1 ml of LB, 100 μl of cells was plated onto MH+KAN50 with 64 μg/ml nourseothricin. After incubation at 37°C overnight a colony was picked into LB+KAN50 and used as the template for colony PCR to confirm the insert. The LB+KAN50 culture was used to prepare a −80°C stock and the sequence of the methyltransferase containing contig was confirmed by Sanger sequencing.

The nourseothricin resistance of E. coli expressing the entire methyltransferase contig, a pZE21-ME empty vector strain, and three strains from the Minimal Antibiotic Resistance Platform (ARP) (56) were determined by broth microdilution assay. The three additional strains were E. coli DH5α with plasmids pGDP3-*rmtB* (expressing the *rmtB* 16S rRNA methyltransferase gene), pGDP4-*ermC* (expressing the *ermC* 23S rRNA methyltransferase gene), and pGDP1-*stat* (expressing the *stat* streptothricin acetyltransferase gene). The ARP was a gift from Gerard Wright (Addgene kit no. 1000000143) (56). A broth microdilution assay was performed in 200-μl volumes of MH broth in 96-well plates with nourseothricin concentrations ranging from 1,024 μg/ml to 0.5 μg/ml (final concentrations). Wells containing 100 μl volumes of 2× concentrated antibiotic were inoculated in quadruplicate with 100-μl suspensions of each strain at an optical density of 0.5 McFarland standards. Plates were incubated at 37°C with shaking overnight. MICs were determined by eye, and 50% inhibitory concentrations (IC_50_s) were determined by reading optical density at 600 nm and fitting the resulting dose-response curves to a four-parameter Hill equation using GraphPad Prism version 7.01 (GraphPad Software, La Jolla, CA, USA). The average IC_50_s for the contig-expressing and empty vector E. coli strains were compared for significance using a paired two-tailed *t* test.

### Literature search for comparable library statistics.

A literature search was carried out in the National Library of Medicine using PubMed with the search terms “functional metagenomics” OR “metagenomic libraries” on 30 September 2020. The results were sorted by publication date, and the 125 most recent publications were manually examined for functional metagenomic library preparation details, including insert size (small or large), input DNA mass, and total library size. An additional four publications known to contain these details, including a representative library prepared using restriction enzyme digested inserts, were appended to the 125 publications to give a total of 129 publications searched. Off-topic publications (*n* = 46) that matched key words but did not report preparing or using functional metagenomic libraries, publications that did not describe the preparation of new libraries because they either were review articles (*n* = 17) or reused previously generated libraries (*n* = 10), publications with insufficient methods details, such as lacking explicit quantification of input metagenomic DNA (*n* = 47), and publications passing the above filters but reporting preparation of large-insert functional metagenomic libraries (*n* = 2) were removed, leaving seven suitable publications: references 21, 28, 35, 44, 45, 51, and 57. Library efficiency was determined by normalization of reported library size to reported or best-estimate input metagenomic DNA mass. Normalization to input metagenomic DNA mass, as opposed to the mass of inserts after fragmentation and size selection, was chosen in order to reflect the entire library preparation process beginning immediately after metagenomic DNA extraction and before DNA fragmentation.

### Data availability.

Raw sequencing files are available in NCBI under BioProject number PRJNA736438 and BioSample numbers SAMN19645604, SAMN19645597, SAMN19645598, SAMN19645363, SAMN19645364, SAMN19644893, and SAMN19644894. Scripts for data analysis and statistics are available at https://github.com/hartmann-lab.
