# Supplementary material for: Mosaic Ends Tagmentation (METa) Assembly for Highly Efficient Construction of Functional Metagenomic Libraries
Source: mSystems. 2021 Jun 29;6(3):e00524-21. doi: 10.1128/mSystems.00524-21 (PMC8269240; doi:10.1128/mSystems.00524-21)
Supplement: TEXT S1 [file msystems.00524-21-s0001.docx]

**SUPPLEMENTAL METHODS**

**Preparation and purification of transposase enzyme**

Expression and purification of transposase enzyme was carried out based on modifications of protocols published by Picelli *et al.* and Hennig *et al.* (41, 42). *E. coli* XL1 blue carrying the pTXB1-Tn5 plasmid was a gift from Rickard Sandberg (Addgene plasmid #60240; <http://n2t.net/addgene:60240>; RRID:Addgene_60240) and maintained as specified. The pTXB1-Tn5 plasmid was recovered from an *E. coli* culture grown in LB+CA100 *via* miniprep kit (New England Biolabs, cat#T1010S). Chemically competent *E. coli* BL21(DE3) cells (New England Biolabs, cat#C2527I) were transformed by the plasmid following manufacturer recommendations and selected on LB+CA100 before maintenance as a 15% glycerol stock at -80℃. A single colony of this strain was used to inoculate 1 ml of LB+CA100 and incubated shaking overnight at 37℃. In the morning, the saturated overnight culture was used to inoculate 1 L of Studier ZYM-5052 auto-induction media (84) in a 2.8 L Fernbach flask. The culture was grown aerobically at 37℃ until it began to turn turbid by eye, approximately 3 hours, at which point the temperature was decreased to 20℃ and shaking maintained at 350 rpm overnight. Cells were collected by centrifugation at 8,000 rcf for 20 min at 4℃ after OD_600_ measurements suggested growth had plateaued at an OD_600_ of ~3.72 AU. The resulting wet cell pellet weighed 8.08 g and a sample was analyzed by SDS-PAGE (sodium dodecyl sulfate polyacrylamide gel electrophoresis) to verify induction of the ~75 kDa Tn5-chitin binding domain fusion protein.

The cell pellet was resuspended to 10% w/v in HEGX buffer composed of 20 mM HEPES buffer pH 7.2, 0.8 M NaCl, 1 mM ethylenediaminetetraacetic acid (EDTA), 10% v/v glycerol, and 0.2% v/v triton X-100 and supplemented with 20 μM phenylmethylsulfonyl fluoride (PMSF) as a protease inhibitor. Resuspended cells were lysed on ice by sonication using a W-225 sonicator with 6 cycles of 1 min on and 1 min off at output 5, 50% duty. Insoluble debris was removed by centrifugation at 15,000 rcf for 30 min at 4℃ following which 2.1 ml of neutralized 10% polyethyleneimine (Millipore Sigma, cat#P3143) was added to the decanted supernatant dropwise while stirring at 4℃. The precipitated *E. coli* genomic DNA was removed by centrifugation for 10 min at 9,000 rcf at 4℃.

The fusion protein was purified from the clarified supernatants by adding 20 ml of chitin resin (New England Biolabs, cat#S6651S) and incubating with gentle rotation overnight at 4℃. The resin was washed with approximately 400 ml of HEGX buffer, following which the drained resin was added to 30 ml of HEGX buffer supplemented with 100 mM β-mercaptoethanol to initiate intein cleavage of the Tn5 transposase from the chitin binding domain. Cleavage proceeded with gentle rotation at 4℃ for approximately 48 hr after which transposase was collected from the resin by draining and saving the flow-through. The resin was washed with 2X dialysis buffer consisting of 100 mM HEPES pH 7.2, 0.2 M NaCl, 0.2 mM EDTA, 20% w/v glycerol, 0.2% triton X-100, and 2 mM dithiothreitol (DTT) and washes were pooled with the initial elution. The pooled eluates were concentrated and exchanged into 2X dialysis buffer using an Amicon Ultra 15 ml 3,000 molecular weight cut-off filter (Millipore Sigma, cat#UFC900324). This concentrate contained 1.85 mg/ml protein as determined by BCA assay (Thermo Scientific, cat#23225) in *ca.* 5.67 ml 2X dialysis buffer. After adding 6.2 ml of glycerol and 1.89 ml 2X dialysis buffer enzyme stocks were stored at -20℃ as 500 μl aliquots. Final transposase stocks contained approximately 763 ng/μl protein.

**Verification of transposase activity**

Enzyme activity was verified by observing degradation of a soil metagenomic DNA extract. Mosaic end primers 5Phos_METagA1 and METagA2 (**Supplemental table 1**) synthesized by Integrated DNA Technologies (IDT Inc.) were brought to a concentration of 100 μM in 50 mM NaCl, 40 mM Tris pH 8. Annealing was carried out by combining 10 μl aliquots of each oligo and incubating in a thermocycler using the following settings: 5:00 at 95℃, cool to 65℃ at 0.1℃/sec, hold at 65℃ for 5:00, and cool to 4℃ at 0.1℃/sec. Annealed oligos were maintained as aliquots at -20℃ until use. Loaded transposomes were prepared by combining 0.143 volumes of annealed mosaic ends oligos with one volume of 763 ng/µl transposase stock and incubating at room temperature (*ca.* 23℃) for 1 hr.

As a test substrate, metagenomic DNA was extracted from a soil sample taken from the Northwestern University campus (coordinates 42.05662, -87.674247) using a DNeasy PowerSoil Kit (Qiagen, cat#12888-100). Test tagmentation reactions were performed by combining MilliQ water (to 20 μl final volume), 4 μl of 5X TAPS-DMF buffer (50 mM TAPS buffer pH 8.5, 25 mM MgCl_2_, 50% v/v dimethylformamide), 1 μl of 50 ng/μl soil metagenomic DNA, and 1 μl of water (control) or 1 μl of loaded transposome (corresponding to 665 ng). Reactions were incubated in a thermocycler for 7 min at 55℃ at which point reactions were quenched by addition of 5 μl of 0.2% SDS (final concentration 0.05% SDS) and incubation at 55℃ for 5 min. For each 25 μl quenched reaction (+/- transposome), 12.5 μl was purified using a silica column-based kit and eluted with 12.5 μl water while the remaining 12.5 μl were not purified further. Next, 2.5 μl of 6X loading dye was added to both the un-purified reactions and purified reactions and samples were analyzed on an agarose gel. Conversion of the high molecular weight DNA smear into a low molecular weight smear confirmed active transposase.
