# Supplementary material for: Mosaic Ends Tagmentation (METa) Assembly for Highly Efficient Construction of Functional Metagenomic Libraries
Source: mSystems. 2021 Jun 29;6(3):e00524-21. doi: 10.1128/mSystems.00524-21 (PMC8269240; doi:10.1128/mSystems.00524-21)
Supplement: TEXT S2 [file msystems.00524-21-s0002.docx]

**SUPPLEMENTAL RESULTS**

**Expression, purification, and testing of transposase enzyme**

We modified published protocols by Picelli *et al*. and Hennig *et al.* for expression and purification of Tn5 transposase enzyme, most notably in our use of auto-induction media (84) and other expression conditions. The expression system responded well to auto-induction (**Supplemental figure 5A**) with one liter of culture yielding 8 g of wet cell mass and we obtained approximately 10.5 mg of well purified enzyme (**Supplemental figure 5B**) in good agreement with reported yields of 15 mg per liter of culture (41). We verified the activity of our transposase by observing its ability to convert metagenomic DNA into low molecular weight fragments <500 bp in size (**Supplemental figure 5C**).
