# Supplementary figures and images for: Mosaic Ends Tagmentation (METa) Assembly for Highly Efficient Construction of Functional Metagenomic Libraries
Source: mSystems. 2021 Jun 29;6(3):e00524-21. doi: 10.1128/mSystems.00524-21 (PMC8269240; doi:10.1128/mSystems.00524-21)

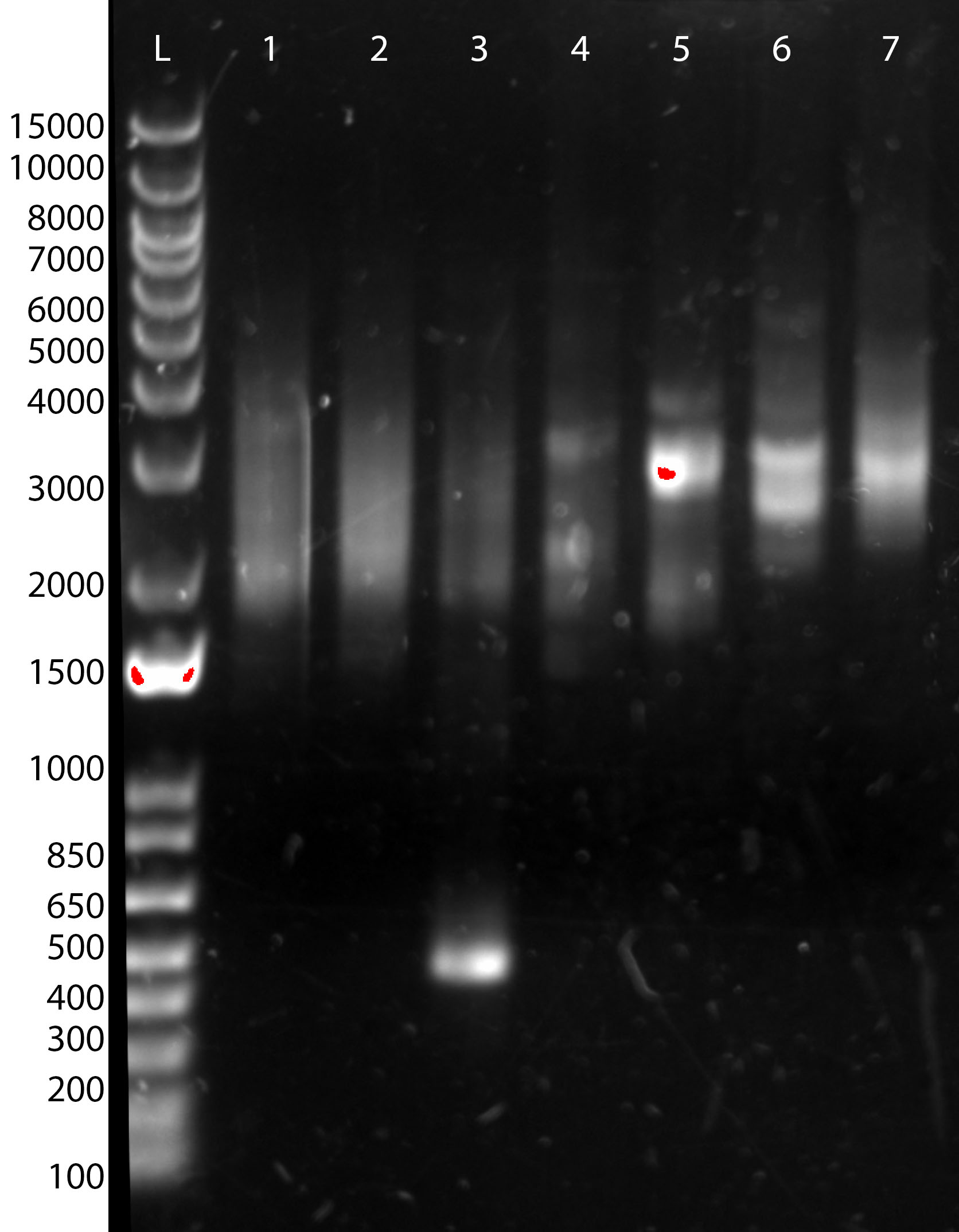

Supplement: FIG S2 [file msystems.00524-21-sf002.jpg]

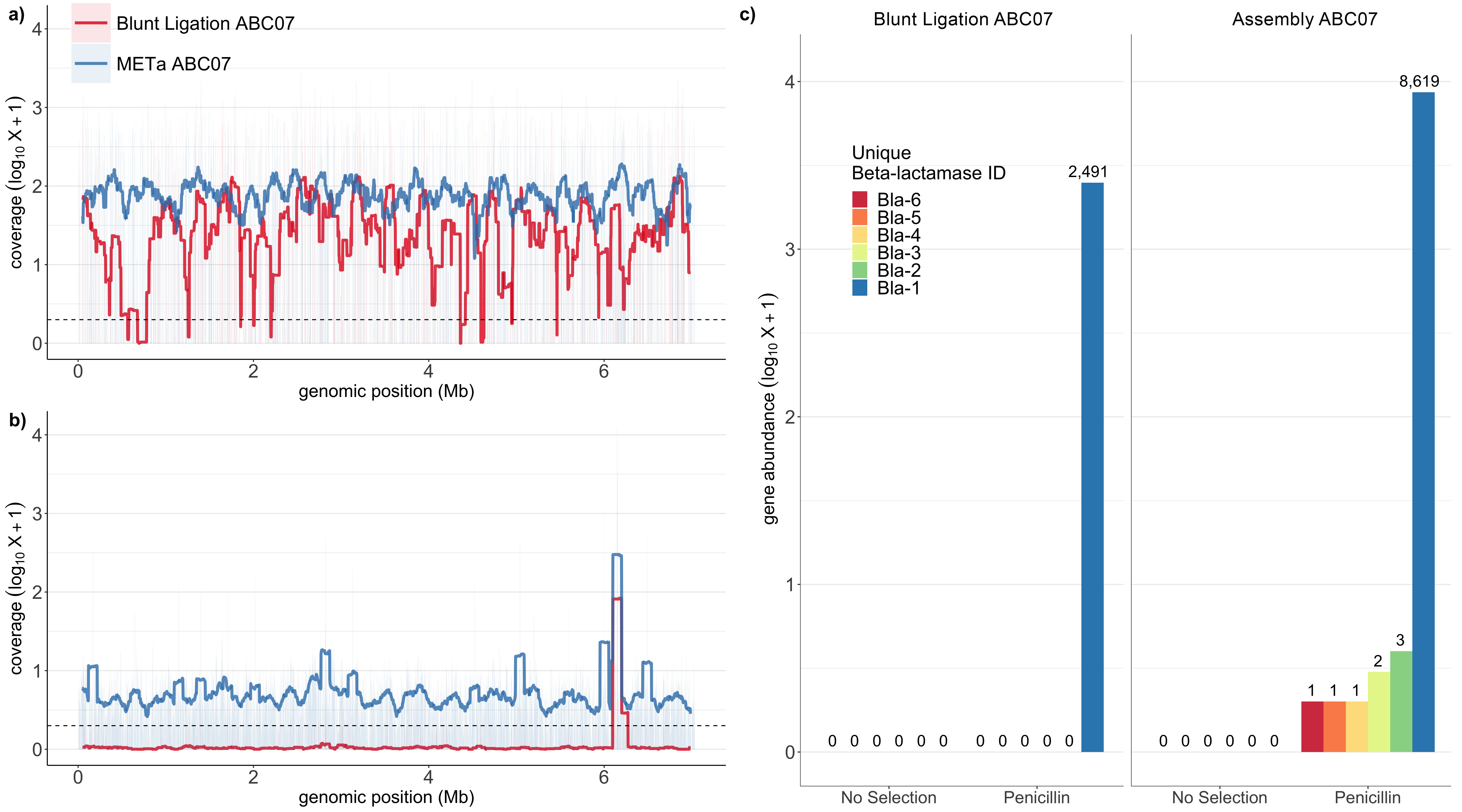

Supplement: FIG S3 [file msystems.00524-21-sf003.jpg]

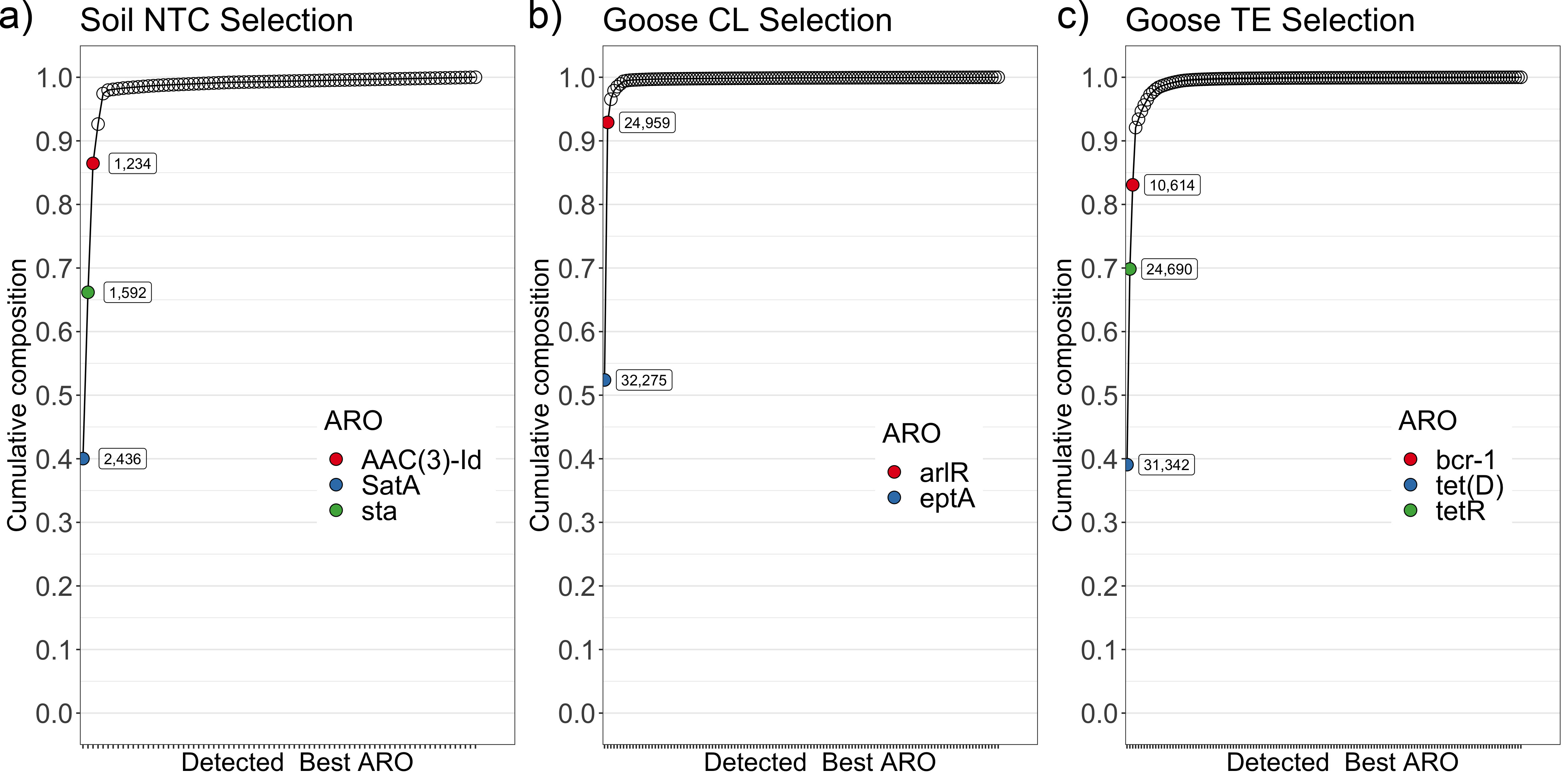

Supplement: FIG S4 [file msystems.00524-21-sf004.jpg]

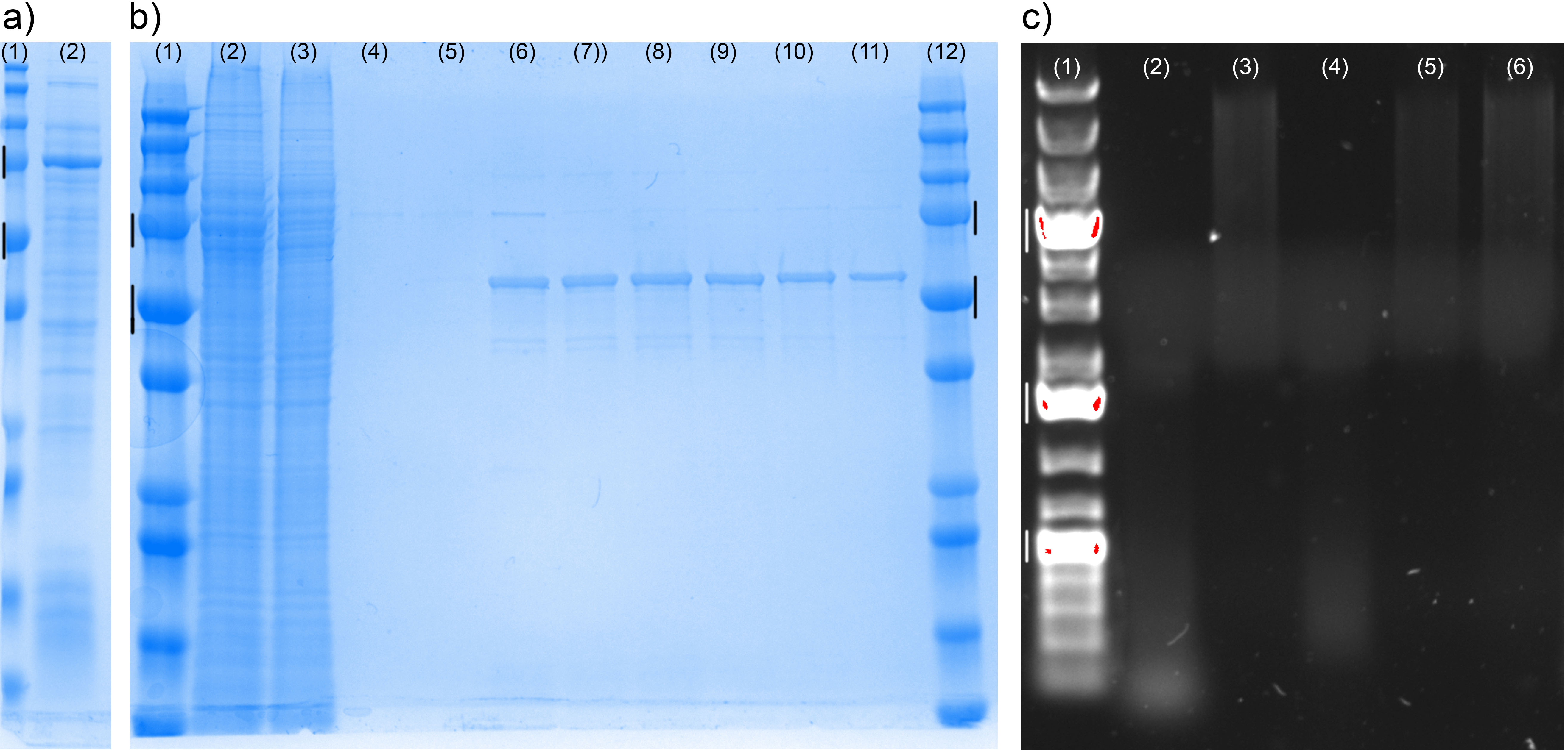

Supplement: FIG S5 [file msystems.00524-21-sf005.jpg]

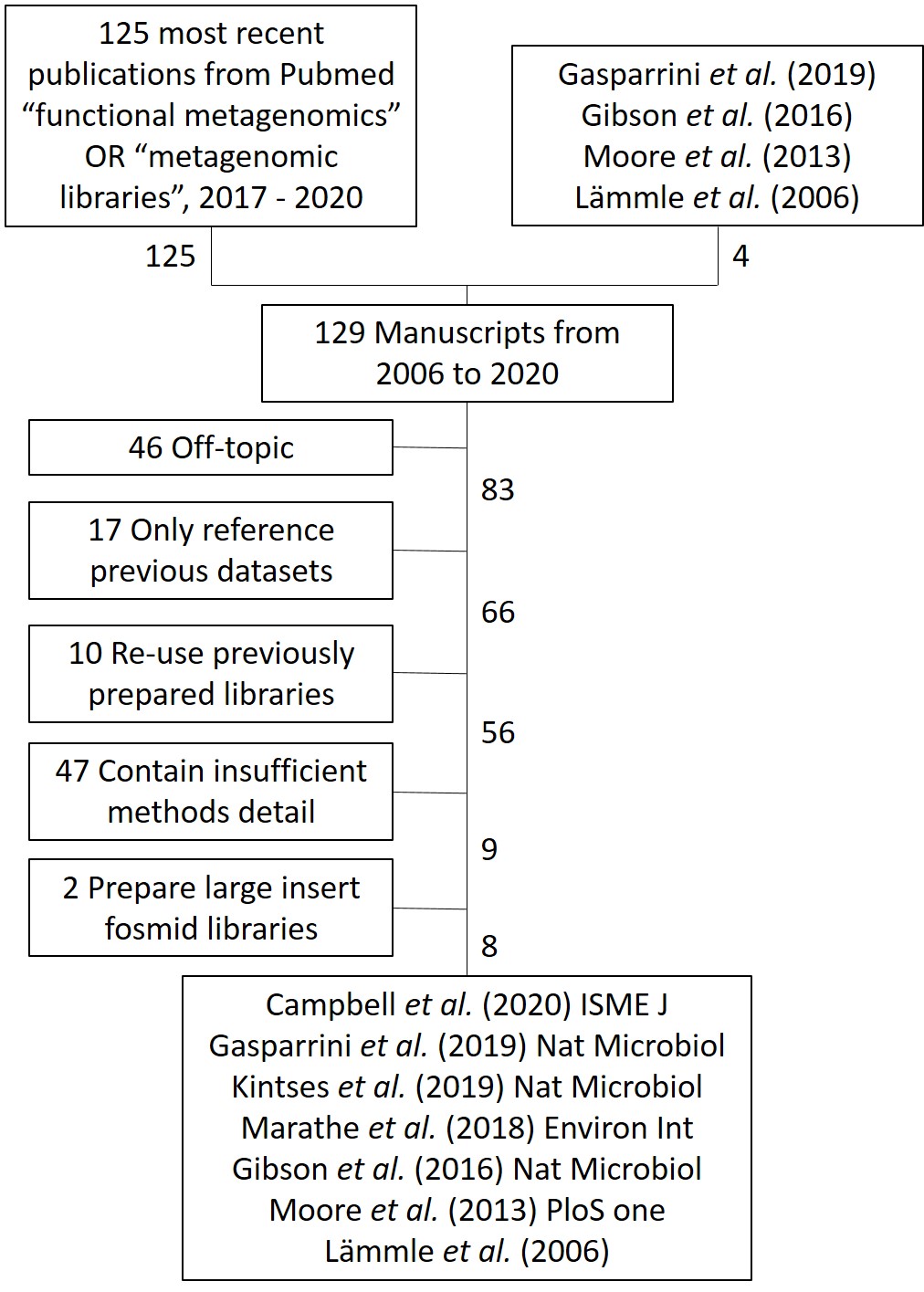

Supplement: FIG S6 [file msystems.00524-21-sf006.jpg]
